# Spontaneous and Naloxone-Precipitated Withdrawal Behaviors From Chronic Opiates are Accompanied by Changes in *N*-Oleoylglycine and *N*-Oleoylalanine Levels in the Brain and Ameliorated by Treatment With These Mediators

**DOI:** 10.3389/fphar.2021.706703

**Published:** 2021-09-15

**Authors:** Samantha M. Ayoub, Fabiana Piscitelli, Cristoforo Silvestri, Cheryl L. Limebeer, Erin M. Rock, Reem Smoum, Mathew Farag, Hannah de Almeida, Megan T. Sullivan, Sébastien Lacroix, Besma Boubertakh, Nayudu Nallabelli, Aron H Lichtman, Francesco Leri, Raphael Mechoulam, Vincenzo Di Marzo, Linda A. Parker

**Affiliations:** ^1^ Department of Psychology and Collaborative Neuroscience, University of Guelph, Guelph, ON, Canada; ^2^ Institute of Biomolecular Chemistry, Endocannabinoid Research Group Consiglio Nazionale delle Richerche, Pozzuli, Italy; ^3^ Centre de Recherche de l’Institut Universitaire de Cardiologie et Pneumologie de Québec, Faculty of Medicine, Centre NUTRISS, Université Laval, Québec City, QC, Canada; ^4^ Institute of Drug Research, School of Pharmacy, Medical Faculty, Hebrew University of Jerusalem, Jerusalem, Israel; ^5^ Department of Pharmacology and Toxicology, Medical College of Virginia Campus Virginia Commonwealth University, Richmond, VA, United States; ^6^ Faculty of Agriculture and Food Science, INAF, Université Laval, Québec City, QC, Canada; ^7^ Canada Excellence Research Chair on the Microbiome/Endocannabinoidome Axis in Metabolic Health, Québec City, QC, Canada

**Keywords:** oleoyl alanine, oleoyl glycine, opiate withdrawal, endocannabinoidome, somatic withdrawal, heroin self-administration, CB_1_, PPARalpha

## Abstract

**Rationale:** The endocannabinoidome mediators, *N*-Oleoylglycine (OlGly) and *N*-Oleoylalanine (OlAla), have been shown to reduce acute naloxone-precipitated morphine withdrawal affective and somatic responses.

**Objectives:** To determine the role and mechanism of action of OlGly and OlAla in withdrawal responses from chronic exposure to opiates in male Sprague-Dawley rats.

**Methods:** Opiate withdrawal was produced: 1) spontaneously 24 h following chronic exposure to escalating doses of morphine over 14 days (Experiments 1 and 2) and steady-state exposure to heroin by minipumps for 12 days (Experiment 3), 2) by naloxone injection during steady-state heroin exposure (Experiment 4), 3) by naloxone injection during operant heroin self-administration (Experiment 5).

**Results:** In Experiment 1, spontaneous morphine withdrawal produced somatic withdrawal reactions. The behavioral withdrawal reactions were accompanied by suppressed endogenous levels of OlGly in the nucleus accumbens, amygdala, and prefrontal cortex, *N*-Arachidonylglycerol and OlAla in the amygdala, 2-arachidonoylglycerol in the nucleus accumbens, amygdala and interoceptive insular cortex, and by changes in colonic microbiota composition. In Experiment 2, treatment with OlAla, but not OlGly, reduced spontaneous morphine withdrawal responses. In Experiment 3, OlAla attenuated spontaneous steady-state heroin withdrawal responses at both 5 and 20 mg/kg; OlGly only reduced withdrawal responses at the higher dose of 20 mg/kg. Experiment 4 demonstrated that naloxone-precipitated heroin withdrawal from steady-state exposure to heroin (7 mg/kg/day for 12 days) is accompanied by tissue-specific changes in brain or gut endocannabinoidome mediator, including OlGly and OlAla, levels and colonic microbiota composition, and that OlAla (5 mg/kg) attenuated behavioural withdrawal reactions, while also reversing some of the changes in brain and gut endocannabinoidome and gut microbiota induced by naloxone. Experiment 5 demonstrated that although OlAla (5 mg/kg) did not interfere with operant heroin self-administration on its own, it blocked naloxone-precipitated elevation of heroin self-administration behavior.

**Conclusion:** These results suggest that OlAla and OlGly are two endogenous mediators whose brain concentrations respond to chronic opiate treatment and withdrawal concomitantly with changes in colon microbiota composition, and that OlAla may be more effective than OlGly in suppressing chronic opiate withdrawal responses.

## Introduction

A new member of the increasing family of endocannabinoid (eCB)-like mediators, which, together with their receptors and metabolic enzymes, constitute the endocannabinoidome (eCBome) (Di Marzo 2018), *N*-Oleoylglycine (OlGly) is an endogenous lipoaminoacid produced in the brain following mild trauma and has been shown to reduce the behavioral consequences of this condition (Piscitelli et al., 2020) as well as the abuse-related effects of nicotine addiction in mouse models (Donvito al., 2019). OlGly also reduced responses produced by acute naloxone-precipitated morphine withdrawal (MWD) in rats as assessed by conditioned place aversion (CPA; Petrie et al., 2019) and somatic withdrawal behaviors (Rock et al., 2020). *In vitro* evidence indicates that OlGly acts as a peroxisome proliferator-activated receptor alpha (PPARα) agonist and interacts with the eCB system through its weak inhibition of the eCB-degrading enzyme, fatty acid amide hydrolase (FAAH) (Donvito et al., 2019), but without directly activating the two major eCB receptors, CB_1_ and CB_2_. While the potential of OlGly to block nicotine conditioned place preference (CPP) in mice was prevented by pretreatment with a PPARα antagonist (Donvito et al., 2019), the attenuation by OlGly with MWD-induced place aversion was prevented by a CB_1_ antagonist, but not by a PPARα antagonist (Petrie et al., 2019). However, OlGly attenuation of somatic MWD behaviors was prevented by both a CB_1_ antagonist and a PPARα antagonist (Rock et al., 2020). Thus, in rats, OlGly reduces acute naloxone-precipitated MWD somatic responses by PPARα- and CB_1_ receptor-dependent mechanisms, whereas its effect on affective responses during withdrawal are mediated exclusively by the CB_1_ receptor, probably *via* its indirect activation due to FAAH inhibition leading to increased levels of endogenous CB_1_ agonists.

As an endogenous amide, OlGly is susceptible to rapid degradation by amidases which may limit its effectiveness as a therapeutic drug for opiate withdrawal. *N-*Arachidonoylethanolamide (anandamide [AEA]), which is a fatty-acid amide eCB with structural similarities to OlGly, was stabilized by Abadji et al. (1994) *via* the addition of a methyl group near the amide linkage to form methanandamide. This compound was more stable against FAAH and possessed greater potency as a ligand for the CB_1_ receptor. Based on this result, we hypothesized that OlGly could be stabilized against amidase degradation in the body by the addition of a methyl group to the glycine’s alpha carbon, sterically hindering enzymatic cleavage at the amide bond. This newly synthesized lipoaminoacid, *N*-Oleoylalanine (OlAla), was indeed shown to block the establishment of affective symptoms of naloxone-precipitated MWD for a longer duration than OlGly, and to mediate its effects *via* CB_1_ and PPARα receptors. Additionally, OlAla was as effective as OlGly in reducing somatic MWD symptoms, including conditioned gaping (a measure of nausea) to a flavor paired with MWD. Consistently, OlAla was found to inhibit FAAH and activate PPARα *in vitro* (Ayoub et al., 2020). Interestingly, evidence exists for the presence of endogenous OlAla in the brain and its lack of significant activity at TRPV1 channels, another target of some eCBome mediators (Raboune et al., 2014).

Recent studies in both rodents and humans have suggested that gut microbiota might be modulated by chronic opiate use and intervene in tolerance to and dependence from opiates (Banerjee et al., 2016; Kang et al., 2017; Wang et al., 2018; Acharya et al., 2017; Gicquelais et al., 2020; see Meckel and Kiraly 2019 for a review). Indeed, the gut microbiota is increasingly being suggested to play a role in behavior and its disturbances induced by psychotropics (see Cussotto et al., 2019 for a review) and to interact with eCBome signaling in both the brain and gut (Manca et al., 2020a; Manca et al., 2020b; see Iannotti and Di Marzo, 2021 for review). Nevertheless, whether or not the gut microbiota is also involved in the behavioral symptoms of opiate withdrawal, and the effects thereupon of eCBome mediators, such as OlGly and OlAla, is not known yet. A recent paper that appeared during the revision process of this manuscript (Thomaz et al., 2021) showed that fecal microbiota transplantation from morphine-treated mice and antibiotic treatment both attenuate naloxone-precipitated opioid withdrawal, as assessed by measuring the number of jumps induced by naloxone in morphine-dependent mice, thus strongly suggesting that the gut microbiota impacts the development of physical dependence induced by chronic use of opiates.

The current study aims to expand on the previous findings on the role of OlGly and OlAla in opiate withdrawal by using male Sprague Dawley rats undergoing spontaneous and naloxone-precipitated opiate withdrawal. We evaluated if OlGly and OlAla: 1) are present in specific brain areas involved in addiction and withdrawal symptoms; 2) reduce behavioral withdrawal responses and do so in a manner mediated by activation of the CB_1_ receptor and PPARα and correlated with changes in brain eCBome signaling and gut (colon) microbiota composition. In Experiment 1, rats were injected with escalating doses of morphine or saline every 12 h for 14 days. Twenty-four hr after the final injection, spontaneous somatic symptoms were measured. Immediately following testing, animals were sacrificed, and brain tissues were taken from the nucleus accumbens (NAc), prefrontal cortex (PFC), interoceptive insular cortex (IIC) and the amygdala. Within these brain regions, levels of the eCBome mediators, AEA and 2-arachidonoylglycerol (2-AG) (two eCBs acting at CB_1_ and CB_2_ receptors), oleoylethanolamide (OEA), palmitoylethanolamide (PEA) and other *N*-acylethanolamines, OlGly, *N*-arachidonoylglycine (AraGly), and OlAla, among others, were quantified by LC-MS-MS. Colonic samples were also taken at the end of the experiment to look at the possible concomitant changes in gut microbiota composition. Experiment 2 was conducted as Experiment 1, except that the rats were pretreated with Vehicle, OlGly or OlAla 10 min prior to spontaneous or naloxone-precipitated withdrawal. In Experiment 3, rats were implanted with osmotic minipumps containing heroin (because of solubility limits for appropriate doses of morphine *via* minipumps) for 12 days. On day 12, rats were treated with Vehicle, OlGly (5 or 20 mg/kg, i.p.) or OlAla (5 or 20 mg/kg, i.p.) 2, 4, and 24 h following removal of the pumps prior to spontaneous somatic withdrawal testing. In Experiment 4, the effect of OlAla on naloxone-precipitated heroin withdrawal was evaluated on Day 12 in rats with intact osmotic minipumps containing saline or heroin (Exp. 4), with brain and gut samples again evaluated for eCBome mediator levels and microbiota composition, in this case allowing to distinguish between the effects on these endpoints of both chronic treatment with, and withdrawal from, chronic opiate administration. Finally Experiment 5 evaluated the potential of OlAla to interfere with operant heroin self-administration on a Fixed Ratio 1 (FR1) schedule and naloxone-precipitated elevation of heroin self-administration.

## Materials and Methods

### Subjects

A total of 243 male Sprague Dawley rats were acquired at 200–225 g from Charles River Laboratories (Saint-Constant, QC) and began experimental testing at an average weight of 300–325 g (average age of 60–70 days). The rats were pair-housed in Plexiglas chambers and located in a colony room maintained on a 12-h reversed light/dark cycle (7:00/19:00) with lights on at 19:00 h, and a temperature of 21 C. Standard rat chow (Teklad Global Diets, 14% protein rodent diet) and water were provided ad-libitum. Following their arrival, rats were given 1 week of acclimation to the testing facility prior to any experimental procedures. All experimental testing began 2 h into the rat’s dark cycle and were approved by The University of Guelph Animal Care Committee and adhere to the guidelines of the Canadian Council on Animal Care.

### Drugs

Oleoylglycine (OlGly) and Oleoylalanine (OlAla) were provided by the Mechoulam laboratory, and administered intraperitoneal (i.p.) at a dose of 5 and 20 mg/kg in a volume of 1 ml/kg. Both compounds were dissolved in a glass graduated cylinder in ethanol with Tween 80 added to the solution, the ethanol was then evaporated off with a nitrogen stream, after which saline was added (final Tween80: saline ratio = 1:9). Vehicle (1 ml/kg) for cannabinoid compounds consisted of a 1:9 mixture of Tween80 to saline. Naloxone (1 mg/ml) was prepared with physiological saline and administered subcutaneously at a dose of 1 mg/kg.

In Experiment 1 and 2, morphine (5, 10, 15, 20, 25, 30, 35, 40 mg/ml) was prepared with physiological saline and administered subcutaneously (s.c) at a volume of 2 ml/kg to result in the respective doses: 10, 20, 30, 40, 50, 60, 70, 80 mg/kg.

In Experiments 3 and 4, the osmotic minipumps (Alzet Model 2002) used express 0.5 ul/hour for a period of 14 days (total volume = 238 ul). Because animals weighed an average of 300 g over the course of the experiment pumps were filled with a heroin solution (following the Alzet pump instructions) at a concentration of 175 mg/ml to result in a 7 mg/kg dose of heroin daily.

In Experiment 5, Heroin (OVC pharmacy) was prepared in a saline vehicle at a concentration of 4 mg/ml. This stock was diluted in the liquid infusion pumps according to daily weights to ensure rats received doses of 0.05 mg/kg/infusion during self-administration.

### Surgery

All surgical manipulations occurred following acclimatization to the colony room. In Experiments 3 and 4 rats were surgically implanted with osmotic minipumps under isoflurane anesthesia. Briefly, a small incision (approximately 3 cm) was made on the skin beginning approximately 2 cm caudal to the scapulae of the animal. Using a hemostat to spread apart the subcutaneous layer a pocket was formed in a caudal direction. The pump was then inserted into the pocket with the flow moderator pointing caudally and the incision was closed with a wound clip (BD Autoclip Wound Closing System, Fisher Scientific, Toronto, ON, Canada). The animal was closely monitored following surgery until they displayed normal respiration, posture, and locomotor activity.

In Experiment 5, rats were surgically implanted with intravenous catheters under isoflurane anesthesia according to the procedure described by Minhas and Leri (2014). Following catheter implantation rats were monitored for 1 week prior to the first day of operant self-administration.

### Extraction and Quantification of OlGly, AraGly, OlAla, 2-AG, AEA and Other *N*-Acyl-Ethanolamines, and *N*-Acylserotonin

Brain and intestinal tissues were frozen in liquid nitrogen immediately after dissection, which took place within 5 min from sacrifice. Tissues were dounce-homogenized and extracted with chloroform/methanol/Tris-HCl 50 mM pH 7.5 (2:1:1, v/v) containing internal deuterated standards for AEA, 2-AG, PEA, OEA, AraGly, OlGly, and OlAla and *N*-acylserotonin quantification by isotope dilution (5 pmol for [2H]8AEA; 50 pmol for [2H]52-AG, [2H]4 PEA, [2H]2 OEA; 10 pmol for [2H]4 DHEA and [2H]4 EPEA; 50 pmol for [2H]8AraGly and [2H]2OlGly; 100 pmol for [2H]8OlAla; 50 pmol for [2H]8 AA5HT). Then, the lipid extract was dissolved in 100 ul of CH_3_OH and analyzed by either LC-APCI-MS for AEA, 2-AG, PEA, OEA, DHEA, and EPEA quantification, which were calculated based on their area ratio with the internal deuterated standard signal areas (Bisogno et al., 1997; Piscitelli et al., 2020), and by LC-MS-IT-TOF (Shimadzu Corporation, Kyoto, Japan) for *N*-acylglycine, *N*-oleoylalanine and *N*-acylserotonin identification and quantification, using multiple reaction monitoring (MRM). The chromatograms of the high-resolution [M-H]- values were extracted and used for calibration and quantification. LC analysis was performed in the isocratic mode using a Phenomenex Kintex Polar C18 column (50 × 3 mm, 2.6 μm) and CH3OH/water/formic acid (85:15:0.1 by vol.) as the mobile phase with a flow rate of 0.15 ml/min. Identification of *N*-acylglycines, *N*-acylserotonins and *N*-oleoylalanine was carried out using ESI ionization in the negative mode with nebulizing gas flow of 1.5 ml/min and curved desolvation line temperature of 250°C. OA5HT was quantified using the peak of deuterated AA5HT as internal standard. The quantification was performed by isotope dilution by using m/z values of 340.2836 and 338.2672 corresponding to the molecular ion [M-H]- for deuterated and undeuterated OlGly, respectively; m/z values of 368.3027 and 360.2830 corresponding to the molecular ion [M-H]- for deuterated and undeuterated AraGly, respectively; m/z values of 356.3159 and 352.2835 corresponding to the molecular ion [M-H]- for deuterated and undeuterated OlAla, respectively; 469.3310 and 461.3319 corresponding to the molecular ion [M-H]- for deuterated and undeuterated AA5HT, respectively; or m/z value of 439.2873 corresponding to the molecular ion [M-H]- of undeuterated OA-5-HT. The most dominant product ion for each lipid class (m/z 74 corresponding to glycine loss, [M-H]^−^ m/z 88 corresponding to alanine loss, [M-H]^−^, or 161 corresponding to serotonin loss, [M-H]-) was selected for MRM.

At least 10 mediators were measured for each of the analysed six tissues belonging to either 2 or 8 experimental groups, for Experiment 1 and 4, respectively, thus generating at least between 120 and 480 measures, and a huge amount of comparisons to discuss. For this reason, we decided not to show mediator data for which we found no statistically significant differences among groups, nor the corresponding *p* values, which were all higher than 0.05.

### Analysis of Gut Microbiota Composition

DNA was extracted from intestinal contents using the QIAmp PowerFecal DNA kit (Qiagen, Hilden, Germany) according to the manufacturer’s instructions. The DNA concentrations of the extracts were measured fluorometrically with the Quant-iT PicoGreen dsDNA Kit (Thermo Fisher Scientific, MA, United States) and the DNAs were stored at −20°C until 16S rDNA library preparation. Briefly, 1ng of DNA was used as template and the V3-V4 region of the 16S rRNA gene was amplified by polymerase chain reaction (PCR) using the QIAseq 16S Region Panel protocol in conjunction with the QIAseq 16S/ITS 384-Index I (Sets A, B, C, D) kit (Qiagen, Hilden, Germany). The 16S metagenomic libraries were eluted in 30 µl of nuclease-free water and 1 µl was qualified with a Bioanalyser DNA 1000 Chip (Agilent, CA, United States) to verify the amplicon size (expected size ∼600 bp) and quantified with a Qubit (Thermo Fisher Scientific, MA, United States). Libraries were then normalized and pooled to 2 nM, denatured and diluted to a final concentration of 6 pM. Sequencing (2 × 300 bp paired-end) was performed using the MiSeq Reagent Kit V3 (600 cycles) on an Illumina MiSeq System. Sequencing reads were generated in less than 65 h. Image analysis and base calling were carried out directly on the MiSeq. Data was processed using the DADA2 pipeline (Callahan et al., 2016) and assignation to bacterial taxa was obtained using the Silva v132 reference database. Sequences present in fewer than 10 samples were filtered out and bacterial abundances were normalized using Cumulative Sum Scaling (CSS, MetagenomeSeq R package) (Paulson et al., 2018). Microbiota composition assessed by calculating alpha and comparison of Bray-Curtis beta-diversity indexes using PERMANOVA were computed using *phyloseq* and *vegan* R packages.

Hundreds of bacterial taxa were measured for each of the analysed 2 intestinal tissues, belonging to 2 or 8 experimental groups, for Experiment 1 and 4, respectively, thus generating hunderds of measures, and an incredible amount of comparisons to discuss. For this reason, we decided not to show taxa for which we found no statistically significant differences among groups, nor the corresponding *p* values, which were all higher than 0.05.

### Apparatus

Somatic withdrawal behaviors were recorded in an observation chamber (22.5 cm × 26.0 cm × 20.0 cm) constructed with four black opaque Plexiglas walls and a lid. This chamber sat on top of a clear glass table with a video camera (Panasonic WV-CP484) placed below to record the ventral surface of the rat. Videos were sent to a computer containing The Observer Software (Noldus Information Technology Inc. Leesburg, VA) which was used to score somatic behaviors.

In Experiment 5, Plexiglas operant conditioning chambers (model ENV-008CT, Med Associates, Georgia, VT) were used for self-administration procedures. Each chamber was located within a larger sound-attenuating partition, with a built-in fan to mask noise and provide ventilation. An overhead house light (28 V) was situated on the back wall of each chamber. The front wall contained a retractable lever (8 cm above floor level) which was situated directly below a cue-light (28 V; 11 cm above floor level) as well as a stationary lever (8-cm above floor level). The retractable, “active”, lever was linked to a liquid infusion pump (Razel Scientific Instruments, Stanford, Connecticut) that was set to deliver a 150 μl/kg heroin infusion over a 5 s interval following a lever-press. The stationary lever served as the “inactive lever” and produced no consequence when manipulated. Stainless steel bars (30.5 cm × 24.1 cm × 21.0 cm) provided the foundation of the conditioning chambers. A MED-PC interface controlled all conditioning chambers and recorded the number of infusions received, and responses emitted on the active and inactive lever.

### Behavioral Procedures

#### Experiment 1: Somatic and Neurochemical Spontaneous Withdrawal Responses Following Chronic Escalating Morphine

A total of nine rats were assigned to receive chronic s.c. exposure to escalating doses (10, 20, 30, 40, 50, 60, 70, 80 mg/kg) of morphine (*n* = 5) or saline (*n* = 4) for 14 days. On the first day of chronic injections, rats were given 10 mg/kg morphine (AM/PM). The dose was then progressively increased every 2 days (i.e., 20 mg/kg on Day 2 and 3, 30 mg/kg on Day 4 and 5, etc.) until receiving one final AM 80 mg/kg dose on Day 14.

Twenty-four hr following the final chronic injection on Day 15, all rats were injected with saline and 10 min later were placed in the somatic withdrawal observation chamber for 30 min to evaluate the behavioral and neurochemical effects of spontaneous withdrawal from chronic morphine. The withdrawal behaviors assessed were abdominal contractions, diarrhea, wet dog shakes, mouth movements and suppressed locomotor activity (Defined in Table 1).

**TABLE 1:** MWD reactions assessed.

Behavior	Type	Definition
Abdominal Contractions	Frequency	The abdomen constricting inwards
Wet Dog Shakes	Frequency	Shaking of fur from head to toe
Diarrhea	Yes/No	Watery, non-solid feces
Mouthing Movements (reflecting chewing)	Frequency	Increased moving mouth from side to side
Locomotor Activity	Duration	Suppressed movement of two paws along horizontal axis of the floor

Immediately following the somatic withdrawal assessment, the rats were euthanized by decapitation and brain (NAc, amygdala, PFC and IIC) and gut tissues (colon) were removed, flash frozen and stored at −80°C for subsequent eCBome analysis by LC-MS-MS.

#### Experiment 2: Effect of OlGly and OlAla on Spontaneous Somatic Withdrawal Responses Following Chronic Escalating Morphine

A total of 96 rats evaluated the potential of OlGly (5 mg/kg, i.p.) and OlAla (5 mg/kg, i.p.) to reverse withdrawal reactions 24 h following from chronic morphine or saline exposure. Half of the rats were tested following an injection of saline and half were tested following an injection of naloxone (s.c.). Twenty-four hr following the final chronic saline or morphine injection on Day 15, the rats were injected with VEH, 5 mg/kg OlGly or 5 mg/kg OlAla 10 min prior to an injection of saline or naloxone and 10 min later were placed in the somatic withdrawal chamber for 30 min during which the rats were videotaped and later scored for withdrawal behaviors. The groups (*n* = 8/group) were: Chronic Saline -VEH-Saline, Chronic Saline-OlGly Saline, Chronic Saline-OlAla-Saline, Chronic Saline VEH-Naloxone, Chronic Saline OlGly-Naloxone, Chronic Saline OlAla-Naloxone, Chronic Morphine -VEH-Saline, Chronic Morphine OlGly Saline, Chronic Morphine-OlAla-Saline, Chronic Morphine VEH-Naloxone, Chronic Morphine OlGly-Naloxone, and Chronic Morphine OlAla-Naloxone.

#### Experiment 3: Effect of OlGly and OlAla on Spontaneous Somatic Withdrawal From Chronic (12 days) Steady-State Heroin

A total of 46 rats were in this experiment, 38 of which were implanted with minipumps containing heroin and eight rats were implanted with minipumps containing saline. The pumps remained intact for 12 days. Twenty-four hr following removal of the pumps, the rats were placed in somatic withdrawal observation chambers (for 30 min) following a pretreatment injection of VEH, OlAla (5 mg/kg or 20 mg/kg, i.p.) or OlGly (5 mg/kg or 20 mg/kg, i.p.). The groups (*n* = 7–8/group) are designated by the pretreatment injection at the time of withdrawal testing and the chronic treatment by minipumps (Saline or Heroin): Group VEH-Saline (*n* = 8), Group VEH-Heroin (*n* = 8), Group 5 OlGly-Heroin (*n* = 7), Group 5 OlAla-Heroin (*n* = 8), Group 20 OlGly-Heroin (*n* = 8) and Group 20 OlAla-Heroin (*n* = 7). Rats received the pretreatment injection (VEH, OlGly or OlAla; i.p.) as well 2 and 4 h after removal of the pumps to enhance the clinical relevance by increasing the likelihood of exposure to the treatment with onset of withdrawal.

#### Experiment 4: Effect of OlAla on Naloxone-Precipitated Somatic and Biochemical Responses in Brain and Gut From Chronic (12 days) Steady-State Heroin

A total of 56 rats were implanted with osmotic minipumps filled with saline (*n* = 28) or heroin (*n* = 28). On Day 12, with the pumps intact, the rats were injected with VEH or OlAla (5 mg/kg) 10 min prior to an injection of the withdrawal treatment drug, saline or naloxone. Ten min after the final injection the rats were placed in the somatic withdrawal observation chambers for 30 min. Immediately after removal from the somatic withdrawal chambers, the brain and gut tissue was collected and stored at −80°C as in Experiment 1. The groups (*n* = 7) were: Chronic saline-VEH-saline, Chronic saline-OlAla-saline, Chronic heroin-VEH-saline, Chronic heroin-OlAla-saline, Chronic saline-VEH-naloxone, Chronic saline-OlAla-naloxone, Chronic heroin-VEH-naloxone and Chronic heroin-OlAla-naloxone.

To determine the mechanism of action of the effect of OlAla on naloxone-precipitated heroin withdrawal, an additional two groups (*n* = 8/group) were implanted with heroin minipumps and on Day 12, were given the CB_1_ antagonist, AM251 (1 mg/kg, i.p.), or PPARα antagonist, MK886 (1 mg/kg, i.p.), 20 min prior to OlAla (5 mg/kg; i.p.) 10 min prior to naloxone. The doses of these compounds used have been previously demonstrated to have no effect on their own on naloxone induced opiate somatic withdrawal responses (Rock et al., 2020). The rats were then immediately placed in the observation chamber for 30 min and videotaped.

#### Experiment 5: Effects of OlAla on Heroin Self-Administration and Naloxone Potentiation of Heroin Self-Administration

##### Acquisition of Heroin Self-Administration

A total of 20 rats completed the experiment. All self-administration sessions ran daily for 180 min and began 2 h into the rat’s dark cycle. Intravenous catheters were flushed with saline prior to each session to ensure there were no obstructions in the drug delivery line. Once placed in conditioning chambers rats were habituated for 5 min prior to each test session. Illumination of the house light signaled the beginning of a session, with the active lever extending out after a 10 s delay. A continuous schedule was used to reinforce responding on the active lever in which rats received a 150 μl/kg intravenous infusion of heroin (0.05 mg/kg/infusion) and the presentation of a cue-light for 5 s. During this time, additional responses on the active lever were recorded but did not lead to additional reinforcement. Responding on the inactive lever had no programmed consequences and served as a baseline measure of non-reinforced operant responding. The acquisition phase of self-administration lasted for 10 sessions. For the first 3 days, animals were primed (up to 6 primes per session) if no active lever response was emitted within a 15 min period. During sessions 7–10, rats were adapted to the injection procedure with the i.p. administration of 1 ml/kg of VEH 10 min prior to 1 ml/kg s.c saline 5 min prior to being placed in the conditioning chambers. Rats in both groups were maintained on this injection schedule throughout the experiment except during test sessions when the drug group received the appropriate treatment. Following acquisition, rats were assigned to VEH (*n* = 9) or OlAla (*n* = 11) matched for infusions earned and responses (active and inactive) made on the last day of acquisition (session 10). Two baseline sessions were interspersed between each test session to allow self-administration behavior to stabilize following treatment.

##### Effect of OlAla on Heroin Self-Administration

On Session 11 all rats in the drug group (*n* = 11) received OlAla (5 mg/kg, i.p.) and rats in in the VEH group (*n* = 9) received VEH 10 min prior to s.c. saline, 5 min prior to being placed in the conditioning chambers to test whether OlAla itself modified heroin self-administration.

##### Effect of OlAla on Naloxone-Precipitated Withdrawal Induced Elevation of Heroin Self-Administration

On sessions 14 and 17, rats in the drug group received VEH-naloxone and OlAla-naloxone in a counterbalanced order. They were injected with VEH or OlAla (5 mg/kg, i.p.) 10 min prior to naloxone (1 mg/kg, s.c.) 5 min prior to placement in the conditioning chambers.

### Data Analysis

In Experiment 1, the somatic withdrawal behaviors and the *ex vivo* levels of OlGly, AraGly, AEA, 2-AG, OEA, and PEA were analyzed by independent t tests for the chronic saline and chronic morphine group. In Experiment 2, the effect of the pretreatment on the withdrawal behavioral responses were analyzed a 2 (chronic treatment) × 3 (pretreatment) × 2 (test treatment) between groups analysis of variance (ANOVA). In Experiment 3, each of the somatic withdrawal behaviors scored was entered into a one-way ANOVA for groups with subsequent comparisons by Bonferroni post hoc tests. In Experiment 4, the number or duration of each of the behavioral measures were entered into a 2 × 2 × 2 factorial design, with the between group factors of chronic treatment (saline or heroin), withdrawal treatment (saline or naloxone) and pretreatment (VEH or OlAla). To determine the mechanism of action of the interference with naloxone-precipitated heroin withdrawal, the relevant behavioral withdrawal measures for each of the additional two groups (MK886-OlAla and AM251-OlAla) was compared with group heroin-naloxone-OlAla in a single factor ANOVA. Variations in brain biochemicals and fecal microbiota compositions were evaluated using one-way analysis of variance (ANOVA) and Tukey HSD post-hoc tests to assess treatment effects (VEH vs drug groups). For Experiment 5, the self-administration acquisition period, sessions 1–10, the number of infusions received were entered into a repeated measures ANOVA. The number of responses made on the active lever and the inactive lever were entered into a 2 × 10 repeated measures ANOVA with lever (active or inactive) and session (sessions 1–10) as within subject factors. To evaluate the effect of OlAla alone on heroin self-administration on Day 11, an independent t-test was conducted between the VEH and drug group for each self-administration behavior. The VEH-Naloxone and OlAla-Naloxone data for each measure on Days 14 and 17 were pooled, because the order of testing did not differ for any measure as assessed by paired t-tests. The self-administration behaviors for Group VEH, were each entered into a paired t-test across the two test days (Days 14, 17), which revealed no significant differences; therefore, the data for this group was pooled across these days for comparison with the drug group on each test. For the drug group each test measure was then entered into a paired t-test (VEH-Naloxone, OlAla -Naloxone). Finally, each measure for the VEH group was compared with the drug group on all tests. Statistical significance was defined as *p* < 0.05. Box plots borders represent the first and third quarter while the intersecting line represents the median of data. Whiskers representing 1.5× the interquartile range. Samples falling beyond this range are drawn as dots.

## Results

### Experiment 1: Spontaneous Somatic Withdrawal Behaviors and Neurochemical Changes in the Prefrontal Cortex, Amygdala, Nucleus Accumbens and Interoceptive Insular Cortex Following Chronic Escalating Orphine

Rats displayed somatic withdrawal responses 24 h following chronic escalating doses of morphine. These behaviors were accompanied by a suppression of endogenous OlGly in the NAc, amygdala and PFC, as well as suppressed AraGly in the amygdala and suppressed 2-AG in the NAc, amygdala, and IIC.

**FIGURE 1:**
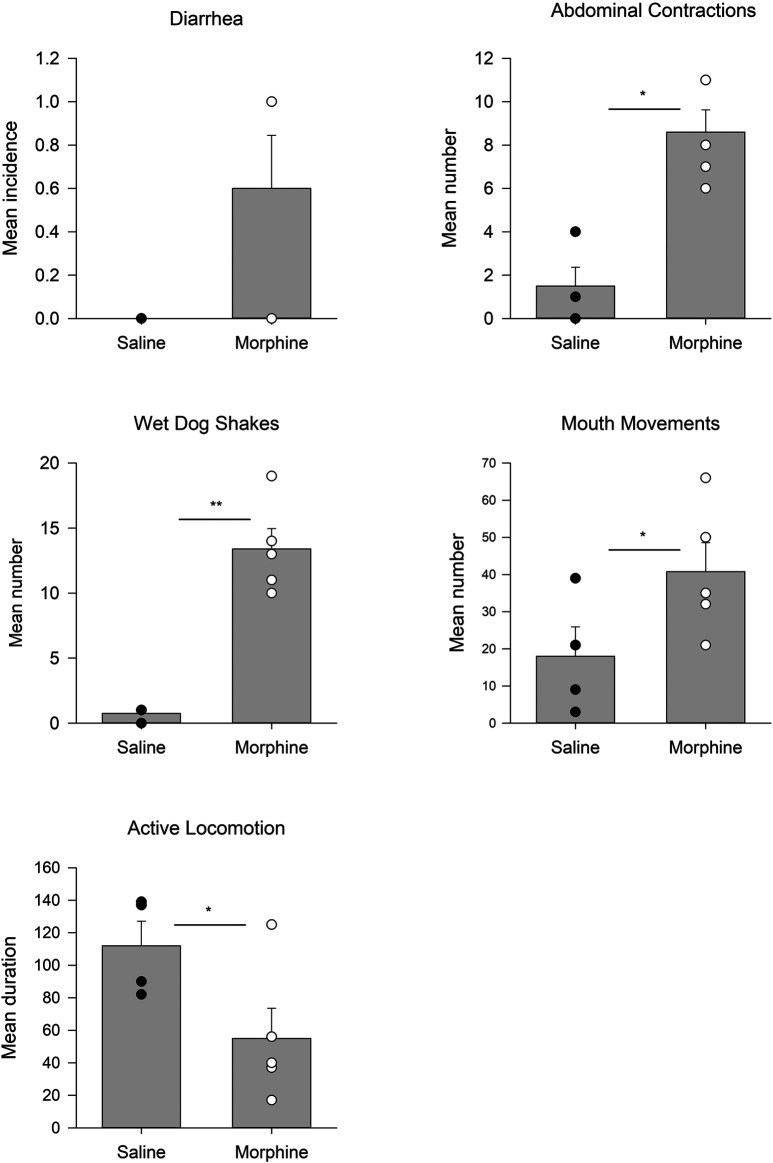
Experiment 1. Mean (±SEM) incidence of diarrhea, number of abdominal contractions, wet dog shakes, mouth movements, and duration of active locomotion displayed by rats 24 h following 14 days of chronic exposure (sc) to saline (*n* = 4) or morphine (*n* = 5). *p < 0.05; **p < 0.01.

Figure 1 presents the somatic behaviors displayed by rats 24 h following chronic escalating doses of morphine or saline in Experiment 1. Rats in the morphine group displayed enhanced abdominal contractions, t(7) = 2.6; *p* = 0.034, enhanced wet dog shakes, t(7) = 3.1; *p* = 0.016, elevated mouthing movements, t(7) = 2.4; *p* < 0.05, and suppressed active locomotion, t(7) = 2.4; *p* = 0.046, relative to rats in the chronic saline group. The groups did not differ in instances of diarrhea.

**FIGURE 2:**
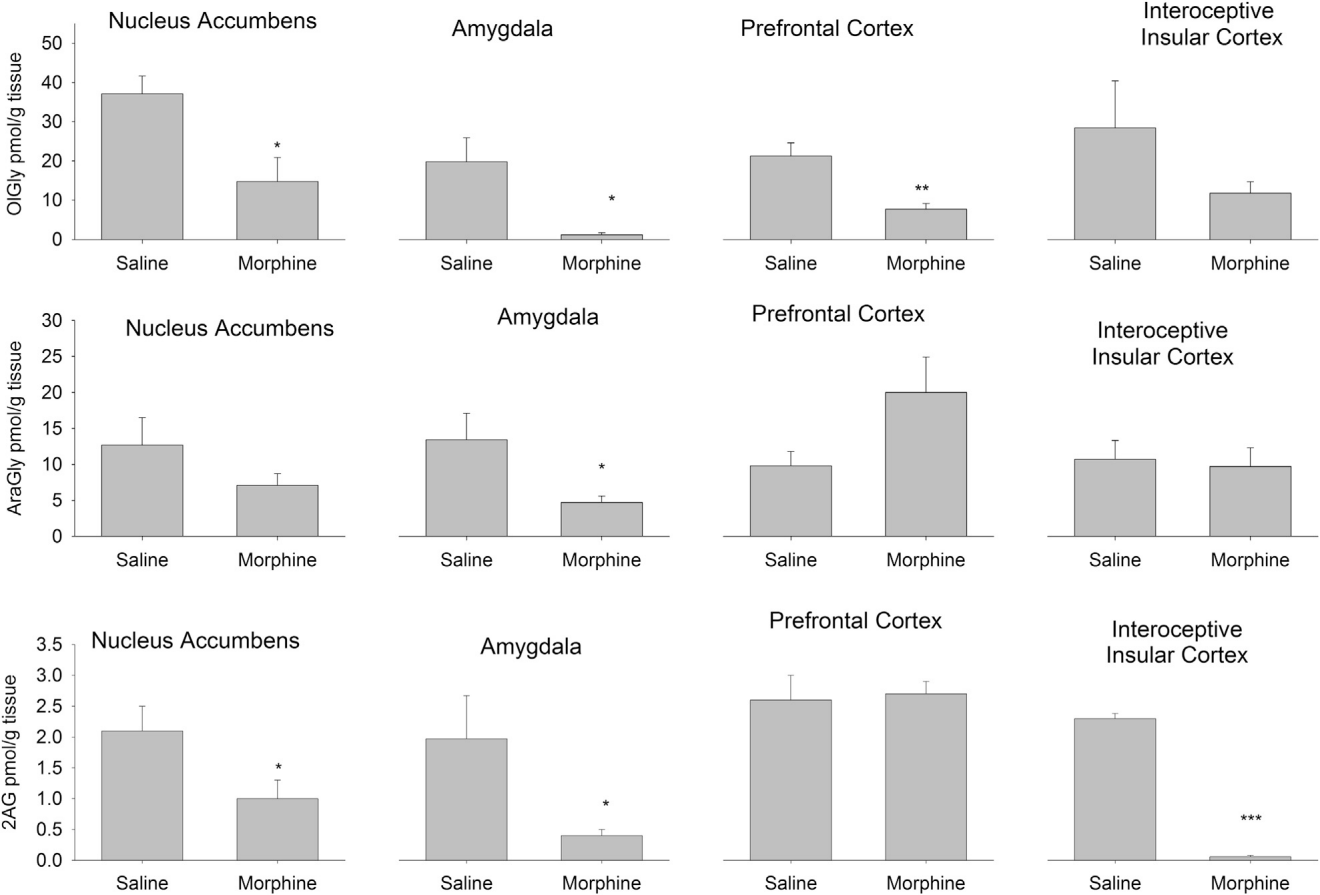
Experiment 1. Quantification of the levels of the eCBome lipid mediators OlGly (top), AraGly (middle) and 2-AG (bottom) in the nucleus accumbens, amygdala, prefrontal cortex, and interoceptive insular cortex of rats 24 h following 14 days of chronic exposure (sc) to saline (*n* = 4) or morphine (*n* = 5). *p < 0.05; **p < 0.01; ***p < 0.001. Data is expressed as mean +SEM.

Figure 2 presents the effects of the administration of escalating doses of morphine followed by a 24 h withdrawal period on endogenous OlGly, AraGly and 2-AG levels in the NAc, amygdala, PFC and IIC. Animals that received chronic morphine had significantly reduced levels of OlGly in the NAc, t(7) = 2.8; *p* = 0.028, amygdala, t(7) = 3.0; *p* = 0.02, and PFC, t(7) = 3.9; *p* = 0.006, but not in the IIC. Morphine treated rats also showed significantly reduced AraGly in the amygdala, t(7) = 2.6; *p* = 0.037 and reduced 2-AG in the NAc, t(7) = 2.4; *p* = 0.048, amygdala, t(7) = 2.9; *p* = 0.0379, and the IIC, t(7) = 29.0; *p* < 0.001. Chronic morphine followed by a 24 h withdrawal period did not modify levels of AEA, OEA or PEA in any region.

We also measured the levels of endogenous brain OlAla, which was detected only in the amygdala. Interestingly, OlAla was significantly suppressed in this brain area (Group saline mean 150 pmol/g tissue [±27.1], Group morphine mean 16.0 pmol/g tissue [±3.7]) of rats undergoing chronic morphine administration followed by a 24 h withdrawal period, t(7) = 5.6; *p* < 0.001).

**FIGURE 3:**
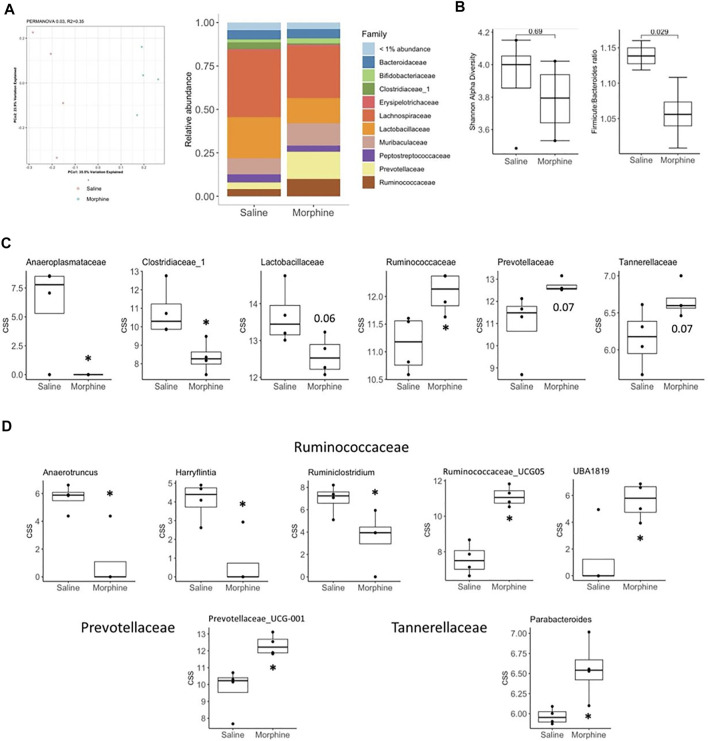
Experiment 1. Microbiome analysis in rats 24 h following 14 days of chronic exposure (sc) to saline (*n* = 4) or morphine (*n* = 4). **(A)** Principal coordinate analysis of bacterial 16S rDNA sequencing results (left) and relative abundance of bacterial families (right) from saline and morphine treated rats. **(B)** Shannon alpha-diversity index (left) evaluating gut microbiota richness and evenness and Firmicutes to Bacteroidetes phyla ratio (right). **(C, D)** cumulative sum scaled abundances (CSS) of bacterial families **(C)** and genera **(D)** found to be found to be modified by morphine. Box plot data is expressed as the median (line) and upper and lower quartiles (boxes) with whiskers indicating samples within 1.5× the interquartile range. Samples falling beyond this range are denoted as dots. *p < 0.05. *p* values above 0.05 are indicated.

We then went on to determine if the rats had significant changes within gut microbiota of their colons by 16S sequencing. Principal Coordinate Analysis followed by PERMANOVA revealed that the morphine treatment resulted in a distinct bacterial community from the control and could also be observed when the relative abundances of bacterial families were assessed (Figure 3A). While no differences were observed within the Shannon alpha diversity index, morphine significantly decreased the *Firmicutes:Bacteroides* ratio (Figure 3B). The differences in bacterial communities appears to be driven by a decreased abundance of the *Anaeroplasmataceae*, *Clostridiaceae_1* and *Lactobacillaceae* families and an increase in *Ruminococcaceae*, *Prevotellaceae* and *Tannerellaceae* (Figure 3C). We were only able to detect significant morphine effects on the abundance of genera within the later families that showed increased abundance (Figure 3D). Within *Prevotellaceae* and *Tannerellaceae*, only *Prevotellaceae_UCG-001* and *Parabacteroides*, respectively, were found to be increased suggesting that these genera drive the changes observed in their respective families. However, within the increasing *Ruminococcaceae* family, in morphine-treated rats, the most prevalent genus *Ruminococcaceae_UCG-005* as well as *UBA1819* were increased, while the *Anaerotruncus*, *Harryflintia* and *Ruminiclostridium* genera were decreased.

### Experiment 2: Effect of OlGly and OlAla on Spontaneous Somatic Withdrawal Responses Following Chronic Escalating Morphine

**FIGURE 4:**
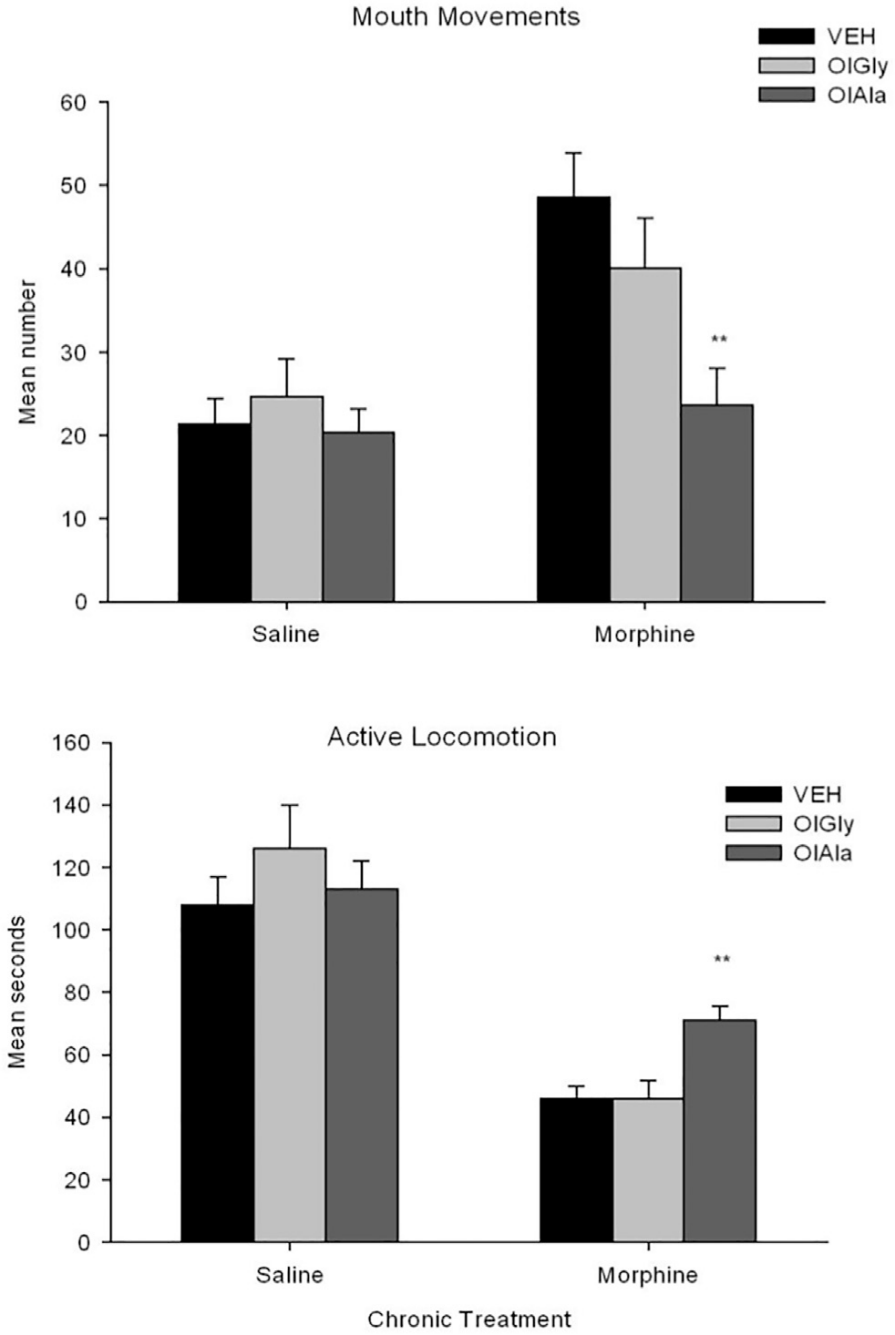
Experiment 2. Mean (±SEM) number of mouthing movements and duration of active locomotion displayed by rats 24 h following chronic exposure (sc) to saline or morphine.nRats were treated (ip) with VEH (*n* = 16), OlAla (5 mg/kg; *n* = 16) or OlGly (5 mg/kg; *n* = 16) 10 min prior to a saline or naloxone (data is pooled across saline and naloxone tests). **p < 0.01 from Vehicle group.

OlAla, but not OlGly, blocked mouthing movements, and attenuated the locomotor deficits produced by withdrawal from chronic morphine, when pooled across naloxone and saline test groups. The behaviors of diahrrea and abdominal contractions did not reveal significant effects of pretreatment. Figure 4 shows the mean number of mouthing movements and seconds of active locomotion displayed by the various groups pooled across naloxone and saline test treatment groups in Experiment 2. The 2 × 3 × 2 factorial ANOVA for mouth movements revealed a significant effect of chronic exposure, F (1, 84) = 19.7; p, 0.001 and chronic exposure x pretreatment, F(2, 84) = 3.8; *p* = 0.027. There was no effect of test treatment. Overall, rats displayed more mouthing movements in the chronic morphine groups than the chronic saline groups. Pooled across the saline and naloxone tests, rats chronically treated with saline did not significantly differ by pretreatment, but rats chronically treated with morphine displayed a significant pretreatment effect, F (2, 47) = 6.5; *p* = 0.003. Rats treated with OlAla displayed fewer mouth movements than rats treated with VEH (*p* < 0.01) and OlGly (*p* = 0.04) by Bonferroni tests.

For active locomotion, the 2 × 3 × 2 factorial ANOVA revealed a significant effect of chronic exposure, F(1, 84) = 99.9; *p* < 0.001, test treatment, F(1, 84) = 23.3; *p* < 0.001, chronic exposure x pretreatment, F(2, 84) = 3.5; *p* = 0.036 and chronic exposure by post-treatment, F(2, 84) = 7.2; *p* = 0.009. Chronic morphine produced suppressed locomotion relative to chronic saline and rats tested with naloxone produced suppressed locomotion relative to saline. Among the chronic saline groups, the pretreatment effect was not significant, but among the chronic morphine groups the pretreatment effect was significant, F(2, 45) = 9.1; *p* < 0.001. Pooled across test drugs, following withdrawal from chronic morphine, rats treated with OlAla were significantly more active than those treated with VEH (*p* < 0.01) or OlGly (*p* < 0.01) by Bonferroni tests. The 2 × 3 × 2 ANOVAs for the additional behaviors (not depicted) also revealed significant effects of chronic treatment for abdominal contractions, F(1, 84) = 40.1; *p* < 0.001) and diarrhea, F (1, 78.7) = 0.001, but neither behavior interacted with the pretreatment variable.

### Experiment 3: Effect of OlGly and OlAla on Spontaneous Somatic Withdrawal From Chronic (12 days) Steady-State Heroin

The only withdrawal behaviors that revealed significant group differences were those of mouthing movements and abdominal contractions. The withdrawal responses of abdominal contractions were suppressed in Groups 5 OlAla-heroin, 20 OlAla-heroin and 20 OlGly-heroin. The withdrawal response of mouthing movements was only suppressed in Group 5 OlAla-heroin.

**FIGURE 5:**
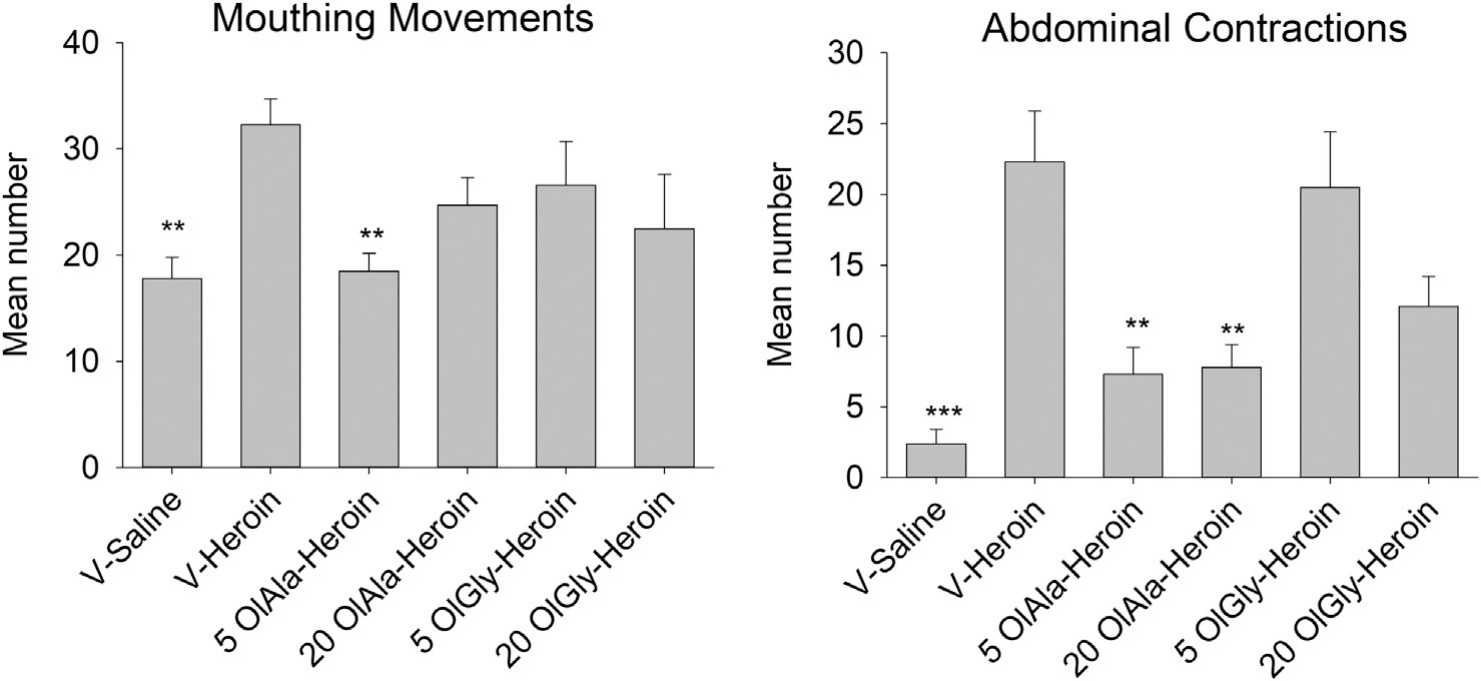
Experiment 3. Mean (±SEM) number of mouthing movements and abdominal contractions displayed by rats 24 h following 12 days exposure to saline or heroin by minipumps across treatment groups (*n* = 7–8/group). Rats were treated with VEH, OlAla (5 mg/kg; 20 mg/kg) or OlGly (5 mg/kg; 20 mg/kg) 2, 4, and 24 h following the removal of minipumps. ***p < 0.001, **p < 0.01.

Figure 5 presents the mean number or duration of each of these behaviors for each group. The one way ANOVAs revealed a significant group effect for mouthing movements, F(5, 40) = 3.1; *p* = 0.05 and abdominal contractions, F(5.40) = 9.2; *p* < 0.001. Subsequent Bonferroni post hoc comparison tests revealed that Group V-heroin displayed significantly more mouthing movements (*p* < 0.01), abdominal contractions (*p* < 0.001) than Group V-saline. As well, Group 5 OlAla-heroin, Group 20 OlAla-heroin displayed fewer (p’s < 0.01) abdominal contractions than Group V-heroin. Group 5 OlAla-heroin also displayed fewer mouthing movements (*p* < 0.05) than Group V-heroin. There was no difference among the groups in duration of active locomotion F(5, 40) = 0.86; *p* = 0.52.

### Experiment 4: Effect of OlAla on Naloxone-Precipitated Withdrawal From Chronic (12 days) Steady-State Heroin and Concomitant Colonic Microbiota and Brain eCBome Mediator Levels

**FIGURE 6:**
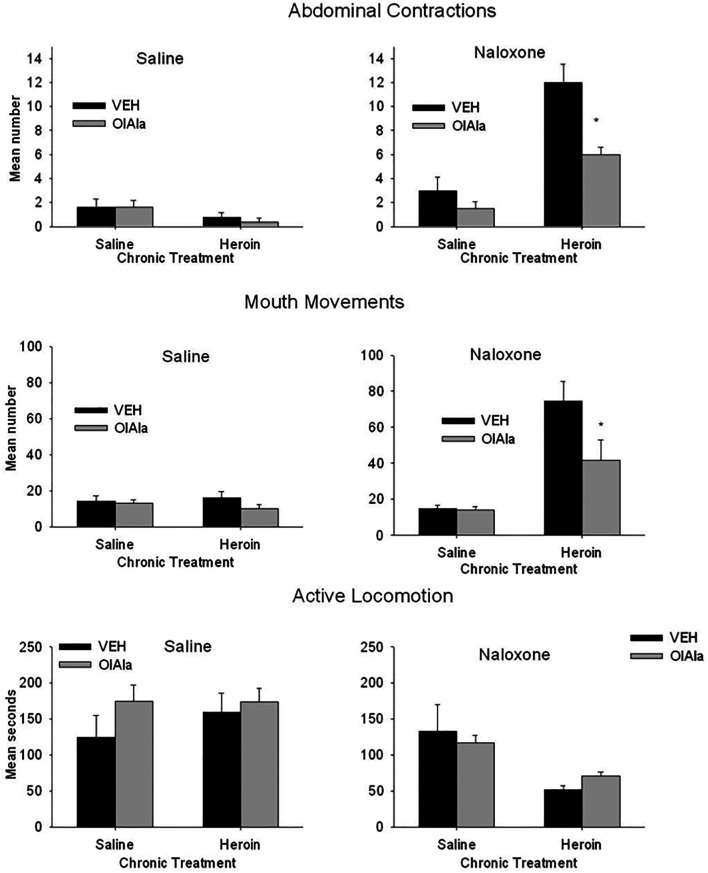
Experiment 4. Mean (±SEM) number of abdominal contractions and mouth movements and duration of active locomotion displayed by rats on Day 12 of chronic exposure to saline or heroin by minipumps across treatment groups (*n* = 8/group). On Day 12 rats were pretreated with VEH or OlAla (5 mg/kg) 10 min prior to saline or naloxone. *p < 0.05, **p < 0.01.

OlAla (5 mg/kg) reduced the naloxone-precipitated withdrawal responses of abdominal contractions and mouth movements, as can be seen in Figure 6. The 2 × 2 × 2 factorial ANOVA for the number of abdominal contractions revealed significant effects of chronic treatment, F(1, 48) = 10.5; *p* = 0.002, withdrawal treatment, F(1, 48) = 80.9; *p* < 0.001, a chronic treatment by withdrawal treatment interaction, F(1, 48) = 22.4; *p* < 0.001, and a chronic treatment by withdrawal treatment by pretreatment interaction, F(1, 48) 11.9; *p* < 0.001. Subsequent analysis of the withdrawal treatment groups separately revealed no significant effects among the saline treated groups, but among the naloxone treated groups there were significant effects of chronic treatment, F(1, 24) = 19.1; *p* < 0.001, and a withdrawal treatment by chronic treatment interaction, F(1, 24) = 9.7; *p* = 0.005. Among the naloxone treated groups, only in the chronic heroin treated rats pretreated with VEH displayed elevated abdominal contractions, OlAla interfered with elevated abdominal contractions (*p* < 0.025).

The 2 × 2 × 2 factorial ANOVA for the response of mouth movements revealed significant effects of chronic treatment, F(1, 48) = 43.4; *p* < 0.001, withdrawal treatment, F(1, 48), 49.3; *p* < 0.001, pretreatment, F(1, 48) = 10.3; *p* = 0.003, a chronic treatment by withdrawal treatment interaction, F(1, 48) = 45.6; *p* < 0.001, a chronic treatment by pretreatment interaction, F(1, 48) = 7.9; *p* = 0.007, a withdrawal treatment by pretreatment interaction, F(1, 48) = 4.1; *p* = 0.048, and a chronic treatment x withdrawal treatment by pretreatment interaction, F(1, 48) = 4.5; *p* = 0.031. Subsequent analysis of the withdrawal treatment groups separately revealed no significant effects among the saline treated groups, but among the naloxone treated group there were significant effects of chronic treatment, F(1, 24) = 54.37; *p* < 0.001, pretreatment, F(1, 24) = 8.2; *p* = 0.008, and a chronic treatment by pretreatment interaction, F(1, 24) = 7.4; *p* = 0.012. Among the naloxone treated groups, those chronically treated with heroin showed more mouth movement when pretreated with VEH than when pretreated with OlAla (*p* < 0.025).

The 2 × 2 × 2 factorial ANOVA for the duration of active locomotion revealed only a significant effect of withdrawal treatment, F(1, 48) = 16.3; *p* < 0.001 and a chronic treatment by withdrawal treatment interaction, F(1, 48) = 9.45; *p* = 0.003. Rats chronically treated with heroin displayed suppressed locomotion relative to those chronically treated with saline despite pretreatment conditions; OlAla did not modify this naloxone-precipitated withdrawal reaction. There were no other naloxone precipitated withdrawal effects observed with any of the remaining behavioral measures.

Both AM251 and MK886 prevented the interference with naloxone-induced heroin withdrawal abdominal contractions and mouth movements by OlAla (data not graphically depicted). A one way ANOVA among OlAla pretreatment groups (OlAla alone, AM251-OlAla and MK886 OlAla) that were treated with naloxone following chronic heroin revealed a significant effect for both abdominal contractions, F(2, 19) = 5.0; *p* = 0.018, and mouth movements, F(2, 19) = 13.4; *p* < 0.001); subsequent Bonferroni comparison tests revealed that the group pretreated with OlAla alone displayed fewer abdominal contractions (7.2 ± 1.3) and mouth movements (41.6 ± 4.2) than the groups pretreated with either AM251 [abdominal contractions (12.6 ± 1.4); mouth movements [90.1 ± 6.8}) or MK886 (abdominal contractions (14.1 ± 2.0); mouth movements (98.4 ± 12.0)]. We have previously shown that neither antagonist modifies these withdrawal -induced behaviors on their own at these doses (Rock et al., 2020).

**FIGURE 7:**
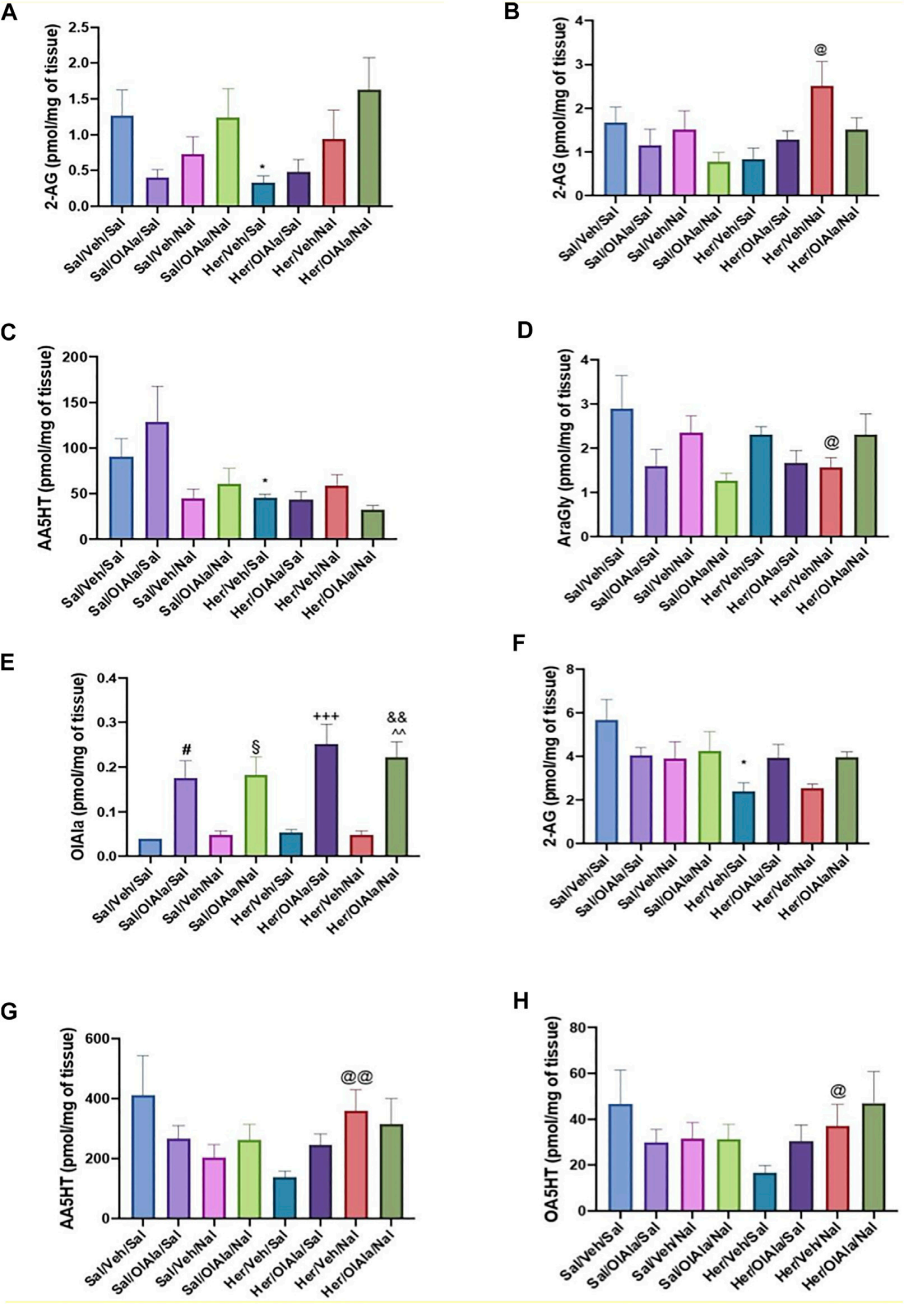
Experiment 4. Quantification of the levels of the eCBome lipid mediators within various tissues of rats following chronic saline or heroin exposure without or with OlAla and/or naloxone precipitated withdrawal. **(A, B)** Levels of 2-arachidonoyl glycerol (2-AG) withn the amygdala **(A)** and prefrontal cortex **(B)**. **(C, D)** Levels of *N*-arachidonoyl-serotonin (AA5HT) **(C)** and *N*-arachidonoylglycine (AraGly) **(D)** within the jejunum. **(E-H)** Levels of *N*-oleoylalanine (OlAla) **(E)**, 2-AG **(F)**, AA5HT **(G)** and *N*-oleoyl-serotonin (OA5HT) **(H)** within the colon. Symbols mark statistical significance (P < 0.05): *; Sal/Veh/Sal vs Her/Veh/Sal; ^#^, Sal/Veh/Sal vs Sal/OlAla/Sal; ^§^, Sal/Veh/Sal vs Sal/OlAla/Nal;+, Her/Veh/Sal vs Her/OlAla/Sal; ^&^, Her/Veh/Sal vs Her/OlAla/Nal; ^^^, Her/Veh/Nal vs Her/OlAla/Nal; ^@^ Her/Veh/Sal vs Her/Veh/Nal. Data is expressed as mean +SEM. *N* = 7.

The effect of chronic heroin and naloxone-precipitated withdrawal therefrom, in the presence or absence of OlAla, on several eCBome mediators (including OlAla, thus providing also an idea of its tissue penetration in OlAla-treated rats) in various brain areas and intestinal sections are shown in Figure 7 and Supplementary Figure 10. Particularly, in the amygdala, 2-AG levels decreased significantly with chronic administration of heroin in comparison to saline, and naloxone with or without concomitant OlAla tended to counteract this effect (Figure 7A, *p* < 0.05). In the PFC, under conditions of naloxone-precipitated heroin withdrawal, 2-AG levels instead increased, and this effect was not observed following administration of OlAla (Figure 7B, *p* < 0.05, Her/Veh/Sal vs Her/Veh/Nal). Other changes in this area did not appear to be strictly related to heroin treatment or naloxone-induced withdrawal *per se*, as heroin reduced AEA levels only in the presence of naloxone, without (*p* < 0.05, Sal/Veh/Nal vs Her/Veh/Nal), or with (*p* < 0.01, Sal/OlAla/Nal vs Her/OlAla/Nal) concomitant OlAla administration (Supplementary Figure 1A). Likewise, in the insular cortex, AEA levels increased in the Her/OlAla/Nal group in comparison to Sal/OlAla/Nal, whereas EPEA levels decreased (Supplementary Figures 10B,C, *p* < 0.001). No significant alterations were observed in the NAc for any of the mediators (data not shown). Perhaps, the most interesting changes were observed in the gut. Particularly, in the jejunum, chronic heroin infusion reduced *N*-arachidonoylserotonin levels (*p* < 0.05) with no subsequent effect of naloxone with or without OlAla (Figure 7C), whereas naloxone-induced precipitated withdrawal was accompanied by reduction in AraGly levels (*p* < 0.05), which was not observed in the presence of OlAla (Figure 7D). The colon was, among the tissues analyzed, the one with most alterations in eCBome mediators. First of all, this was the only tissue where we could observe elevated OlAla levels in rats treated with this compound (Figure 7E). Like in the amygdala, heroin reduced 2-AG levels, but, unlike the amygdala, naloxone counteracted this effect only in the presence of OlAla administration, which was effective also in the absence of naloxone (Figure 7F). AA5HT and OA5HT levels tended to decrease in the colon of heroin-treated rats, and naloxone significantly reversed this effect in a manner not modified by exogenous OlAla (Figures 7G,H). Finally, attenuation of naloxone-precipitated withdrawal signs by OlAla was accompanied by significant (*p* < 0.05) elevation of both AEA and EPEA to levels similar to those observed in rats only treated with OlAla (Supplementary Figures 1D,E), indicating that this effect may not be related to withdrawal but merely to a possible effect of this latter compound on some *N*-acylethanolamines (possibly *via* FAAH inhibition).

**FIGURE 8:**
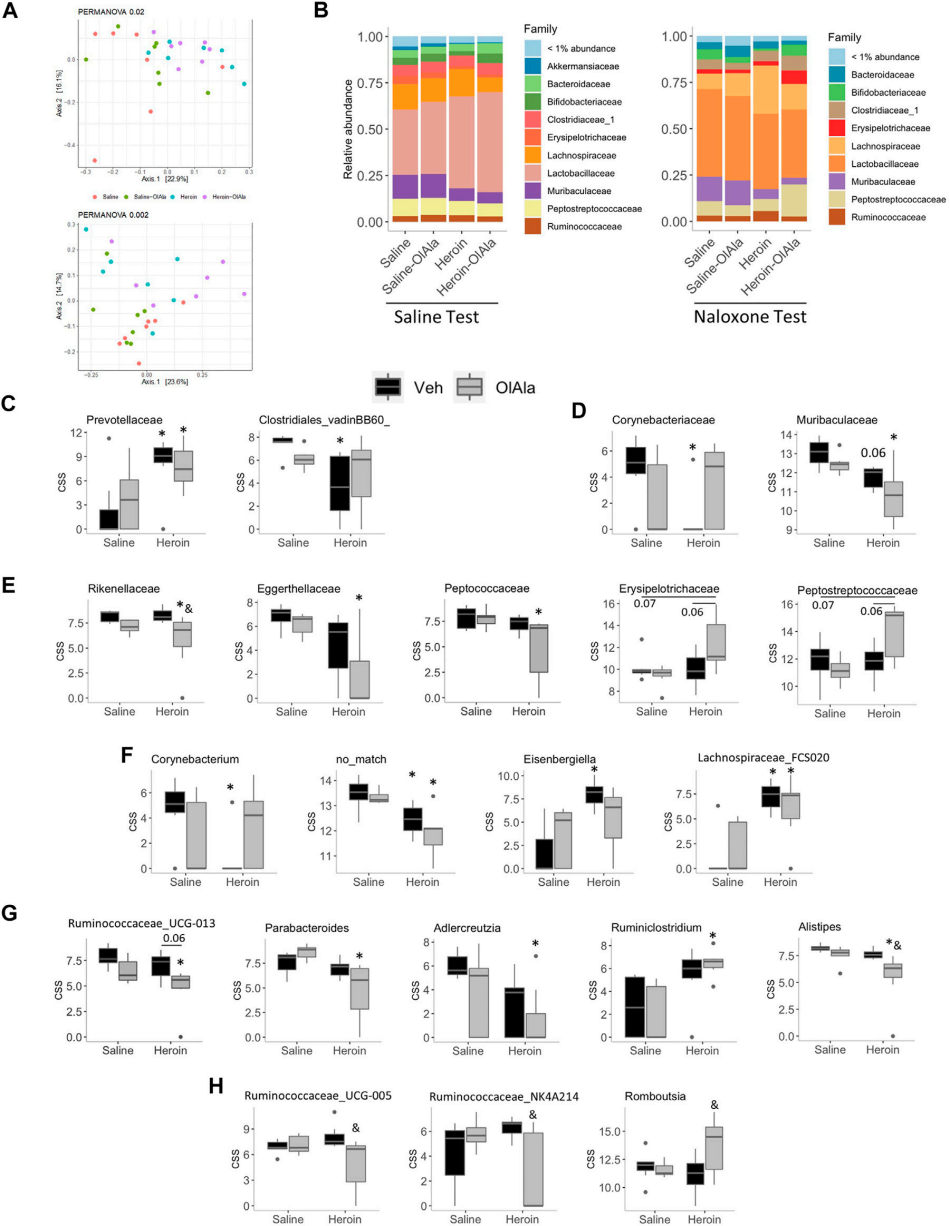
Experiment 4. Microbiome analysis in rats exposed to chronic saline or heroin followed by Saline or Naloxone (Saline or Naloxone tests). **(A)** Principal coordinate analysis of bacterial 16S rDNA sequencing results from Saline (top) or Naloxone (bottom) tested rats **(B)** Relative abundance of bacterial families from Saline (left) and Naloxone (right) tested rats. **(C–G)** Cumulative sum scaled abundances (CSS) of: bacterial families whose abundance was modified by heroin (Veh/heroin vs. Veh/saline) in rats from the Saline test group **(C)**, or naloxone test group **(D)**; bacteria families whose abundance was modified by the combination of heroin and OlAla (OlAla/heroin vs. Veh/saline) in rats from the naloxone test group **(E)**; genera whose abundance was modified by heroin (Veh/heroin vs. Veh/saline) **(F)**, or by the combination of heroin and OlAla (OlAla/heroin vs. Veh/saline) uniquely **(G)** in rats from the naloxone test group; and genera whose abundance was modified by OlAla in heroin treated mice (OlAla/heroin vs. Veh/heroin) within the naloxone test group **(H)**. *p < 0.05 vs. Veh/saline, & *p* < 0.05 vs. Veh/heroin. *p* values above 0.05 are indicated. *N* = 6−7 per group.

Neither acute naloxone nor OlAla (either alone or in combination) had any effect on the colonic microbiota in the absence of heroin administration (data not shown). Heroin appeared to significantly modify the gut microbiota of rats as determined by PCoA analysis followed by PERMANOVA (Saline group *p* = 0.009; Figure 8A, top, naloxone group *p* = 0.002; Figure 8A, bottom), however, post-hoc analysis revealed that only within the naloxone group did heroin modify the microbiota significantly as compared to the relevant control (VEH-saline vs. VEH-heroin; P = 0.03, VEH-saline vs. OlAla-heroin; *p* = 0.03, OlAla-VEH vs. OlAla-heroin; *p* = 0.02). Despite these observed differences, as with morphine, there were no difference in the Shannon alpha diversity indexes between the groups (data not shown), while the *Firmicutes: Bacteroides* ratio was only increased by heroin within the naloxone group (Kruskal-Wallis, *p* = 0.0041), which contrasts with the effect observed above with morphine (data not shown). Bacterial family relative abundance changes in response to chronic heroin were different between saline and naloxone test groups (Figure 8B). Chronic treatment with heroin alone (Saline Test) resulted in an increase in the *Prevotellaceae* and a decrease in *Clostridiales_vadinBB60* families (Figure 8C). While OlAla alone did not affect bacterial family abundance, it partially reverted heroin effects on *Clostridiales_vadinBB60* (rendering the decrease statistically insignificant) without affecting *Prevotellaceae*. Our analysis identified no changes in the abundance of genera belonging to *Clostridiales_vadinBB60*, but the genera *Prevotellaceae_UCG-001* and *Prevotellaceae_Ga6A1* were both increased by heroin (VEH-saline vs. VEH-heroin; *p* = 0.05 for both; data not shown), with OlAla rendering the increase insignificant compared to the control only for the latter.

Surprisingly, the abundance of more bacterial families were altered in rats in the naloxone test group. Heroin alone decreased the abundance of *Muribaculaceace* and *Corynebacteriaceae*, with OlAla treatment rendering the heroin effect insignificant for the later (Figure 8D). While OlAla alone again did not alter the abundance of any family, in conjunction with heroin it decreased the abundance of *Rikenallaceae*, *Eggerthellaceae*, and *Peptococcaceae* as compared to saline controls (Figure 8E). In contrast, OlAla treatment after chronic heroin appeared to have increased abundances of *Erysipelotrichaceae* and *Peptostreptococcaceae* families without affecting their abundance in saline controls (Figure 8E). Thus, specifically under conditions of naloxone-precipitated heroin withdrawal, acute OlAla treatment induced changes of the abundances of a specific set of families. This was similarly found at the genus level, with heroin alone significantly decreasing the abundance of *Corynebacterium* as well as suspected genera with no known match in the taxonomic database while increasing the abundance of *Eisenbergiella* and *Lachnospiraceae_FCS020* (Figure 8F). OlAla administration subsequently negated the significant effects on *Corynebacterium* and *Eisenbergiella*, while having no apparent effect on the other two genera. Interestingly the abundance of a number of genera were only modified in heroin-treated rats that were subsequently given OlAla, with *Ruminococcaceae_UCG-013, Parabacteroides*, *Adlercreutzia* and *Alistipes*, all being decreased as compared to negative controls, while *Ruminiclostridium* was increased (Figure 8G). Of potentially the greatest relevance to OlAla’s ability to alleviate naloxone-mediated heroin withdrawal symptoms, we found a group of genera whose abundances were altered by OlAla as compared to heroin-treated controls, with *Alistipes*, *Ruminococcaceae_UCG-013*, *Ruminococcaceae_UCG-005* (which was increased in rats undergoing morphine withdrawal; see above) and *Ruminococcaceae_NK4A214* being decreased and *Romboutsia* being increased (Figures 8G,H).

### Experiment 5: Effect of OlAla on Naloxone Potentiated Heroin Self-Administration

#### Acquisition of Heroin Self-Administration

The number of infusions received and active lever responses emitted increased across sessions, while the number of inactive lever responses remained consistently low during self-administration acquisition. The analysis revealed a significant effect of Day on infusion received, F(1,19) = 16.9, *p* < 0.001, and subsequent pairwise comparisons determined the number of infusions earned on sessions 5–10 was significantly elevated when compared to session 1 (*p* < 0.01). The analysis of the number of responses made on the active and inactive lever revealed a main effect of session F(9, 171) = 8.6, *p* < 0.001, lever F(1,19) = 52.2, *p* < 0.001 and a significant session × lever interaction F(1,19) = 10.7, *p* < 0.001, with the rats demonstrating an increase in active lever, but not inactive lever responses across sessions (data not depicted).

#### Effect of OlAla Alone on Heroin Self-Administration

Oleoyl alanine alone did not interfere with self-administration of heroin assessed by any measure. Table 2 presents the mean (±SEM) for each measure and there were no significant differences between vehicle and OlAla on Day 11.

**TABLE 2:** Mean (±SEM) frequency of self-administration behaviors for group Vehicle and Drug.

	Total		30 min	
	VEH (*n* = 9)	OlAla (*n* = 11)	VEH (*n* = 9)	OlAla (*n* = 11)
Infusions	30.3 (±5.6)	21.7 (±3.1)	4.9 (±1.1)	4.7 (±0.7)
Active Responses	52.7 (±10.3)	35.5 (±5.4)	14.1 (±5.3)	12.6 (±3.6)
Inactive Responses	3.6 (±2.1)	1.1 (±0.6)	2.2 (±1.7)	0.6 (±0.4)

The groups did not significantly differ on any measure.

#### Effect of OlAla on Naloxone Precipitated Withdrawal Induced Elevation of Heroin Self-Administration

**FIGURE 9:**
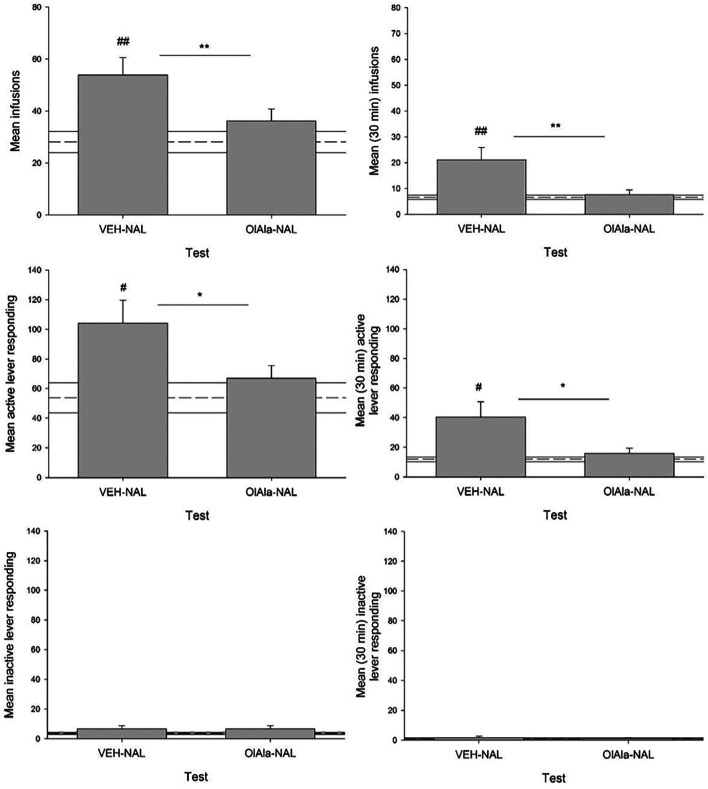
Experiment 5. Mean (±SEM) number of total and first 30 min infusions, active lever responses and inactive lever responses made during a naloxone challenge in rats intravenously administering heroin across test trials (*n* = 9–11/group). Rats were injected with VEH or OlAla (5 mg/kg) counterbalanced order on Days 14 and 17 of heroin self-administration, 10 min prior to naloxone, 5 min prior to placement in the conditioning chambers. Dotted line represents treatment naïve rats. *p < 0.05, **p < 0.01 difference between groups. #*p* < 0.05, ##*p* < 0.01 difference between controls (treatment naïve rats).

Naloxone elevated both the number of infusions and active responses for heroin, effects that were suppressed by pretreatment with OlAla in the pooled tests on Days 14 and 17. Figure 9 presents the mean (±SEM) number of infusions (total and first 30 min), active responses (total and first 30 min) and inactive responses (total and first 30 min) in the pooled VEH-Naloxone and OlAla-Naloxone tests. The mean score (±SEM) of the pooled VEH group is depicted by a dotted line for each measure. Paired t-tests revealed that on the VEH-Naloxone test, rats displayed more total infusions (*p* < 0.01), first 30 min infusions (*p* < 0.01), total active responses (*p* < 0.025) and first 30 min active responses (*p* < 0.025) than on the OlAla-Naloxone test; however, they did not significantly differ in total inactive responses or first 30 min inactive responses. As well, on the VEH-Naloxone test, but not on the OlAla-Naloxone test, naloxone enhanced total infusions (*p* < 0.01), first 30 min infusions (*p* < 0.01), total active responses (*p* < 0.025) and first 30 min active responses (*p* < 0.025) relative to group VEH.

## Discussion

We have provided here data suggesting for the first time that OlGly and/or OlAla, two endogenous lipid mediators belonging to the family of lipoaminoacids of the expanded endocannabinoid system, or eCBome (Di Marzo 2018): 1) are present in brain areas involved in opiate addiction, and their concentrations, like those of other eCBome mediators, are altered following morphine administration and spontaneous withdrawal, or chronic heroin infusion and naloxone-precipitated heroin withdrawal; and 2) when exogenously administered, counteract some somatic signs of withdrawal from chronic opiate exposure, with OlAla being more effective than OlGly, and inducing effects that were dependent on activation of CB_1_ and PPARα receptors and accompanied by changes in gut or brain eCBome mediators and gut microbiota composition. These findings implicate endogenous eCBome mediators and the gut microbiota in the behavioral and neurochemical responses produced by exposure to chronic opiates and withdrawal therefrom, and suggest that administration of OlAla and OlGly *in vivo* attenuates the adverse somatic reactions of opiate withdrawal in rat models.

In Experiment 1, spontaneous withdrawal from chronic escalating morphine injections produced classical somatic behaviors reported in rats following withdrawal from opiates, including reduced locomotion and increased frequency of abdominal contractions, wet dog shakes, and mouthing movements (Rock et al., 2020; Ayoub et al., 2020; Gellert and Holtzman, 1978). These behavioral changes were accompanied by a suppression of: 1) OlGly concentrations in the amygdala, NAc and PFC, 2) AraGly and OlAla concentrations in the amygdala; and 3) 2-AG concentrations in the amygdala, NAc and IIC. However, we cannot conclude that such changes in eCBome mediators, including OlGly and OlAla, were due to administration of escalating doses of morphine or withdrawal from it or both. Clearly, the behavioral measures indicate that the rats experienced MWD immediately prior to tissue collection; however, it is not known if the changes in the eCBome mediators are the result of the withdrawal from opiates or an exposure to opiates over a number of days. There were no changes in any of the measured *N*-acylethanolamines, another eCBome family of mediators. In Experiment 2, pretreatment with 5 mg/kg OlAla, but not 5 mg/kg OlGly, reduced spontaneous somatic withdrawal behaviors (mouthing movements, reduced locomotion) produced by chronic escalating doses of morphine. Taken together, results from Experiments 1 and 2 demonstrate that OlAla is an endogenous compound that can be measured in the amygdala of rats, where its concentrations can be decreased by exposure to morphine and/or withdrawal therefrom, and that systemic administration of this compound attenuates spontaneous withdrawal signs in morphine-dependent rats. The amygdala is a neural substrate involved in opiate addiction (Koob et al., 1992), and Wills et al. (2016) have previously demonstrated that 2-AG regulation in this brain region modifies acute naloxone precipitated MWD induced CPA (affective responses) in rats. Thus, the amygdala could be a target region of OlAla effects on somatic opiate withdrawal behaviours, and, therefore, OlAla should be tested by intracranial experiments in future studies.

The potential of OlAla and OlGly to attenuate withdrawal from chronic opiates was then extended to steady-state heroin *via* minipumps. In Experiment 3, rats displayed increased abdominal contractions and mouthing movements 24 h following the removal of osmotic minipumps that delivered heroin. Pretreatment with high dose OlAla and OlGly (20 mg/kg) reduced abdominal contractions, but only low dose OlAla (5 mg/kg) reduced the abdominal contractions and mouthing movements induced by withdrawal. These dose-dependent effects of OlAla are consistent with the known biphasic effects of eCBome mediators on behaviour (Sulcova et al., 1998; Katsidoni et al., 2013). In Experiment 4, OlAla (5 mg/kg) reduced abdominal contractions and mouthing movements produced by a naloxone challenge in heroin dependent rats, but had no effect on suppressed locomotion. As we have shown with acute naloxone precipitated MWD (Ayoub et al., 2020), the effect of OlAla on naloxone-precipitated withdrawal from chronic heroin was reversed by pretreatment with either the CB_1_ antagonist, AM251, or the PPARα antagonist, MK886, at doses shown not to affect opiate withdrawal behaviors *per se* (Rock et al., 2020). Chronic heroin administration caused a significant decrease in 2-AG concentrations in the amygdala and colon, which were not observed any longer following naloxone administration only in the presence of OlAla. Conversely, naloxone-precipitated withdrawal was accompanied by elevation of 2-AG levels in the PFC, and reduction in AraGly levels in the jejunum, and these effects were counteracted by OlAla. Interestingly, we could not detect OlAla in the brain, even following its administration, whereas this compound was easily detected in the colon, and in amounts significantly higher following exogenous administration. Lastly, in Experiment 5, OlAla (5 mg/kg) did not alter heroin self-administration on its own, consistent with its failure to modify acute morphine conditioned place preference (Ayoub et al., 2020), but eliminated naloxone-induced increases of heroin self-administration behavior. Together these experiments demonstrate that the aversive effects of opiate withdrawal can be reduced by treatment with systemic OlGly and/or OlAla, which could also prevent some of the naloxone-precipitated heroin withdrawal-associated effects on eCBome mediators [and gut microbiota composition (see below)].

The suppression of endogenous OlGly following spontaneous withdrawal from chronic morphine is noteworthy given its previously described neuroprotective properties. In fact, OlGly is elevated in the insular cortex in mice following traumatic brain injury (TBI; Donvito et al., 2019) and systemic administration of this compound reduces the behavioral impairments produced by TBI (i.e., motor, anxiety) (Piscitelli et al., 2020) and nicotine withdrawal (Donvito et al., 2019). We (Petrie et al., 2019) have previously reported that *ex vivo* analysis of tissue from rats experiencing acute naloxone-precipitated MWD revealed that OlGly is elevated in the NAc (but not in the amygdala, PFC and IIC), whereas levels of the endocannabinoids, 2-AG and AEA, and the other endogenous PPARα ligands, OEA, and PEA, were not affected in any of the brain regions evaluated. Therefore, the changes in endogenous levels of OlGly in the NAc following acute administration of morphine and naloxone-precipitated withdrawal therefrom are opposite to those occurring following chronically administered morphine and spontaneous withdrawal therefrom. On the other hand, OlGly suppressed withdrawal responses from acute morphine exposure (Petrie et al., 2019; Rock et al., 2020), but not chronic morphine exposure. It is thus conceivable that the suppression of endogenous OlGly in rats experiencing chronic exposure to morphine, and withdrawal therefrom, may reflect the lack of effectiveness of OlGly to protect against opiate withdrawal in chronically dependent animals. Instead, OlAla, which was not detected in the NAc of rats given morphine under any condition, maintained its effectiveness in reducing opiate withdrawal responses in rats experiencing both acute opiate withdrawal (Ayoub et al., 2020) and chronic opiate withdrawal reported here.

The NAc, amygdala and PFC work collectively to respond to emotionally relevant stimuli (Gruber and McDonald 2012) and are known to be disturbed during opiate withdrawal (Koob et al., 1992; Lyvers and Yakimoff, 2003). The reduction of some eCBome mediators in these brain regions could underlie some other aversive effects of opiate withdrawal. It would therefore be interesting to test whether intracranial administration of OlGly or OlAla in our brain regions of interest would reduce acute or chronic opiate withdrawal behaviors in rats.

The endocannabinoid 2-AG was also suppressed in the NAc and amygdala, and found to be drastically reduced in the IIC. Wills et al. (2016) showed that monoacylglycerol lipase (MGL) inhibition, which increases endogenous 2-AG levels, reduced the establishment of an acute naloxone precipitated MWD-induced CPA, whether administered systemically or directly infused into the IIC and the basolateral amygdala. The IIC monitors internal states and perceptions of well-being (Contreras et al., 2007; Naqvi and Bechara, 2010) and 2-AG in this region has been shown to regulate nausea as measured by conditioned gaping in rats (Sticht et al., 2015; Sticht et al.,2016; Limebeer et al., 2018). Since acute opiate withdrawal produces conditioned gaping reactions in the rat in a manner accompanied by reduced levels of eCBome mediators like OlGly or OlAla, whose systemic administration reduces such reactions (Rock et al., 2020; Ayoub et al., 2020), the reduction of 2-AG in the IIC may also contribute to the nauseous effects of opiate withdrawal that are commonly reported in human addicts. It would be interesting in future studies to investigate if this reduction of 2-AG levels following withdrawal from chronic morphine in rats is due to up-regulation of MGL, and whether the pro-nausea effect of such withdrawal can be counteracted by MGL inhibition.

In the experiments reported here, OlAla was more effective than OlGly in reducing somatic withdrawal responses from chronic exposure to morphine (Ayoub et al., 2020). In Experiment 2, OlAla, but not OlGly, at a dose of 5 mg/kg, i.p., reduced spontaneous withdrawal reactions from chronic exposure to escalating doses of morphine. Likewise, in Experiment 3, OlAla was more effective than OlGly in reducing spontaneous withdrawal reactions from chronic steady-state exposure to heroin by osmotic minipump. As previously mentioned, OlGly belongs to the same lipid family as *N*-arachidonoyl-glycine, which is rapidly degraded by FAAH (Huang et al., 2001). Other *N*-acylated amino acids appear to have lesser affinity as FAAH substrates, although they can still efficaciously inhibit this enzyme (Grazia-Cascio et al., 2004). It is likely that the increased effectiveness of OlAla to reduce opiate withdrawal-related responses is related to its enhanced stability in the body when compared to that of OlGly. Indeed, OlAla produces longer-lasting attenuation of behavioural withdrawal responses then that of OlGly when the two compounds are tested at the same doses, despite the fact that they activate PPARα and inhibit FAAH *in vitro* with similar efficacy when tested at similar concentrations (Ayoub et al., 2020). This suggests that drug stability, rather than potency, contributes to the differences in the behavioral effects produced by OlAla and OlGly in our experiments. Based on this knowledge, and the null effect of OlGly in Experiments 2 and 3, we decided to assess only the effect of OlAla on naloxone-precipitated withdrawal from chronic heroin. In addition to blocking the somatic effects of chronic heroin withdrawal, in a manner antagonized by PPARα and CB_1_ antagonists (in agreement with its previously reported agonist activity at PPARα and inhibition of endocannabinoid inactivation by fatty acid amide hydrolase (FAAH) (Ayoub et al., 2020)), OlAla also reduced naloxone-induced increases of heroin self-administration behavior. It is important to note that OlAla had no effect on heroin self-administration when given on its own, and only altered behaviour following naloxone precipitation. These results complement previous findings that showed that OlGly and OlAla alter opiate withdrawal behaviors while keeping opiate reward processes intact (Petrie et al., 2019; Ayoub et al., 2020). The reduction of operant responding by OlAla during naloxone precipitation can be interpreted in two ways. It is possible that naloxone, as a mu-opioid receptor antagonist, blocked the reinforcing efficacy of self-administered heroin, thereby increasing the rate of responding in rats to achieve reinforcement. Alternatively, it is possible that naloxone-precipitated withdrawal, merely increased the rate of responding in rats to alleviate this adverse state. Given that previous literature highlights the inability of OlGly and OlAla to alter morphine reward (Donvito et al., 2019; Petrie et al., 2019; Ayoub et al., 2020), we lean towards the interpretation that the effect of OlAla on naloxone-induced elevations of heroin self-administration may be a withdrawal-related behaviour. Future work will be needed to address this limitation in our findings.

Additionally, we found that OlAla reversed some of, but not all, the effects of naloxone-precipitated withdrawal from heroin administration on eCBome mediators in both the brain and gut, i.e., it prevented the increase in 2-AG in the PFC and the decrease of AraGly in the jejunum, but not the increase in *N*-acyl-serotonins in the gut. In view of the activity of: 1) 2-AG as an endogenous agonist of PFC CB_1_ receptors potentially involved in anxiogenic responses (Rubio et al., 2008; Llorente-Berzal et al., 2015), 2) AraGly as a FAAH inhibitor and analgesic mediator (Burstein 2018), and 3) *N*-acyl-serotonins as dual blockers of FAAH and TRPV1 (Maione et al., 2007), two targets of anti-emetic and anxiolytic drugs (Sharkey et al., 2007; Micale et al., 2009), we speculate that OlAla might restore only those withdrawal-associated eCBome-mediated adaptive changes that may help counteract withdrawal-induced aversive effects. Indeed, chronic heroin administration also decreased 2-AG concentrations in the amygdala and colon, where this endocannabinoid reduces aversive memories and produces anti-inflammatory actions, respectively (Marsicano et al., 2002; Alhouayek et al., 2011), and this effect was still observed following naloxone administration unless OlAla was co-administered. Therefore, also this action of this lipoaminoacid may have contributed to its beneficial action against naloxone-precipitated heroin withdrawal.

Interestingly, the reduction of *N*-acyl-serotonin levels in intestinal tissues following chronic heroin administration is similar to that observed in mice following 1 week treatment with a cocktail of antibiotics (Guida et al., 2018), and hence is again potentially indicative of the occurrence of opiate-induced intestinal dysbiosis. Indeed, chronic treatment with, and development of tolerance to and addiction from, opiates is now well established to be accompanied by changes in the gut microbiota composition (Banerjee et al., 2016; Wang et al., 2018; Gicquelais et al., 2020; see Salavrakos et al., 2021 for review). Opiates do not need to be administered orally in order for such changes to be observed, and it has been suggested that an altered gut microbiota composition may participate in morphine addiction (Wang et al., 2018). Conversely, morphine tolerance is reversed by probiotics and attenuated in germ-free or antibiotic-treated mice (Zhang et al., 2019), which also exhibit altered endocannabinoid and eCBome signaling in both the gut and brain (Manca et al., 2020a; Manca et al., 2020b). However, except for the very recent paper by Thomaz et al. (2021), which appeared during the revision process of the our manuscript, the role of commensal microorganisms in opiate withdrawal signs had not yet been fully investigated before the present study, and therefore it is not known whether the gut microbiome plays a role in the beneficial effects of eCBome modulating agents, such as OlAla and OlGly, in this context. Here we showed that chronic treatment with, and spontaneous withdrawal from, morphine is accompanied by significant general changes in the colonic microbiome, as shown by: 1) PCoA analysis of the whole dataset, 2) at the phylum ratio, by the *Firmicutes/Bacteriodetes* ratio, which was decreased (Figure 3A), and 3) by the several changes observed at the genus level (Figure 3D), most notably an increase in the relative abundance of *Ruminococcaceae-UCG 005*. Interestingly, the *Firmicutes/Bacteriodetes* ratio is increased in mice undergoing chronic morphine administration through an implanted pellet (Banerjee et al., 2016), whereas the relative abundance of *Ruminococcaceae* is decreased in active opiate users (Acharya et al., 2017). Thus, our findings suggest that morphine withdrawal may result in the reversal of some of the gut microbiota alterations induced by chronic morphine use, and hence that such reversal participates in the signs of withdrawal itself. However, it must be stressed that we did not assess the gut microbiota composition immediately after interruption of morphine administration, but only 24 hs after, i.e., during withdrawal. Thus, the alterations we observed might be the mere result of chronic morphine administration and not of the spontaneous withdrawal. Nevertheless, the aforementioned very recent study by Thomaz et al. (2021) seems to support our hypothesis that the alterations observed were due to withdrawal since the authors showed that also in mice with naloxone-precipitated withdrawal the relative abundance of *Firmicutes* is decreased, similar to our rats during spontaneous withdrawal. Additionally, we reported here that in heroin-treated rats with naloxone-precipitated withdrawal the relative abundance of *Ruminococcaceae-UCG 005* in the colon was reduced by OlAla concomitantly with an amelioration of somatic withdrawal signs (Figure 9), thus suggesting that this genus may be implicated in the behavioral manifestations of heroin, and opiate in general, withdrawal. The family *Ruminococcaceae* has been reported to be instead increased during alcohol dependence (Fan et al., 2018), while fecal microbiota transfer from a donor enriched in this family ameliorated alcohol craving and consumption (Bajaj et al., 2020), thus indicating that members of this family may play different roles in dependence from different substances of abuse. Perhaps more interestingly, inhibition of endocannabinoid signaling has been shown to be accompanied by increased *Ruminococcaceae* abundance in the context of obesity (Chen et al., 2020), possibly in agreement with our present finding that OlAla, a mediator possibly acting in part by elevating endocannabinoid levels *via* FAAH inhibition, instead produces the opposite effect on *Ruminococcaceae-UCG 005*. On the other hand, the morphine-induced microbiome changes reported by Wang et al. (2018) do not mirror those reported here. Notably, that study found that expansion of *Enterococcus faecalis* to be a defining characteristic of morphine induced dysbiosis, and the fact that we were unable to detect any members of the *Enterococcus* genus suggests the existence of baseline differences within the guts of their mice and our rats. Indeed, it is not unusual that different studies, using different species and treatment protocols, lead to the detection, or lack thereof, of certain gut microbiota families, genera or species. In the case of our study and that of Wang et al. (2018) not only species and treatment protocols, but, more importantly, also the sources of the microbiota was different, as we used colon and ileal samples and Wang et al. used fecal samples, where it is not surprising to find a higher abundance of *Enterococcus faecalis*. Accordingly, Thomaz et al. (2021), who used samples from the mouse cecum, also did not detect any *Enterococcus* taxa.

## Conclusion

In conclusion, we have reported here the results of a multi-disciplinary, multi-experiment study aimed at assessing whether chronic morphine or heroin withdrawal behaviors in rats are: 1) accompanied by changes in endocannabinoids and eCBome mediators (belonging to the *N*-acylethanolamine, *N*-acyl-glycine, *N*-acyl-alanine, and *N*-acyl-serotonin classes of lipids) in either the brain or intestine or both; 2) accompanied by changes in intestinal microbiota composition; and 3) counteracted by exogenous administration of the two most abundant endogenous members of the *N*-acyl-glycine and *N*-acyl-alanine lipoaminoacid families in the brain, i.e., OlGly and OlAla. Our findings suggest that OlGly and, more efficaciously, OlAla are capable of reducing somatic signs of opiate withdrawal, that OlAla also counteracts naloxone-precipitated enhancement of heroin self-administration, and that chronic opiate administration and withdrawal therefrom are accompanied by changes in brain and gut eCBome mediator, and gut microbiota, profiles that, in the case of heroin, are also partly reversed by OlAla. Therefore, our study confirms the hypothesis that OlGly and OlAla are endogenous modulators of pathological alterations induced by substances of abuse, and opiates in particular, potentially exploitable as pharmacological treatments for opiate withdrawal syndrome, and capable of exerting their effects by modulating eCBome signaling at either PPARα or CB_1_ receptors, or both, possibly also *via* the increasingly emerging interactions between the eCBome and gut microbiome (Iannotti and Di Marzo, 2021). These findings support previous research that demonstrates the protective role of the CB_1_ receptor in opiate withdrawal (Wills and Parker, 2016), while also highlighting the emerging role of PPARα in this process for the first time. Future research should assess the role of other eCBome mediators as well as of gut microbiome-dervied signals in the behavioral and neurobiological processes involved in opiate withdrawal to advance the neurobiological understanding of opiate withdrawal and to provide new treatment options for human patients.

## Data Availability

The datasets presented in this study can be found in online repositories. The names of the repository/repositories and accession number(s) can be found below: NCBI BioProject, PRJNA742651.

## References

[B1] Abadji V. Lin S. Taha G. Griffin G. Stevenson L. A. Pertwee R. G. (1994). (R)-methanandamide: a Chiral Novel Anandamide Possessing Higher Potency and Metabolic Stability. J. Med. Chem. 37 (12), 1889–1893. 10.1021/jm00038a020 8021930

[B2] Acharya C. Betrapally N. S. Gillevet P. M. Sterling R. K. Akbarali H. White M. B. (2017). Chronic Opioid Use Is Associated with Altered Gut Microbiota and Predicts Readmissions in Patients with Cirrhosis. Aliment. Pharmacol. Ther. 45 (2), 319–331. 10.1111/apt.13858 27868217

[B3] Alhouayek M. Lambert D. M. Delzenne N. M. Cani P. D. Muccioli G. G. (2011). Increasing Endogenous 2-arachidonoylglycerol Levels Counteracts Colitis and Related Systemic Inflammation. FASEB J. 25 (8), 2711–2721. 10.1096/fj.10-176602 21551239

[B4] Ayoub S. M. Smoum R. Farag M. Atwal H. Collins S. A. Rock E. M. (2020). Oleoyl Alanine (HU595): A Stable Monomethylated Oleoyl glycine Interferes with Acute Naloxone Precipitated Morphine Withdrawal in Male Rats. Psychopharmacology (Berl) 237 (9), 2753–2765. 10.1007/s00213-020-05570-4 32556401

[B5] Bajaj J. S. Gavis E. A. Fagan A. Wade J. B. Thacker L. R. Fuchs M. (2020). A Randomized Clinical Trial of Fecal Microbiota Transplant for Alcohol Use Disorder. Hepatology 73 (5), 1688–1700. Baltimore, Md. 10.1002/hep.31496 32750174

[B6] Banerjee S. Sindberg G. Wang F. Meng J. Sharma U. Zhang L. (2016). Opioid-induced Gut Microbial Disruption and Bile Dysregulation Leads to Gut Barrier Compromise and Sustained Systemic Inflammation. Mucosal Immunol. 9 (6), 1418–1428. 10.1038/mi.2016.9 26906406 PMC4996771

[B7] Bisogno T. Ventriglia M. Milone A. Mosca M. Cimino G. Di Marzo V. (1997). Occurrence and Metabolism of Anandamide and Related Acyl-Ethanolamides in Ovaries of the Sea Urchin Paracentrotus lividus. Biochim. Biophys. Acta 1345 (3), 338–348. 10.1016/S0005-2760(97)00009-X 9150253

[B8] Burstein S. H. (2018). N-acyl Amino Acids (Elmiric Acids): Endogenous Signaling Molecules with Therapeutic Potential. Mol. Pharmacol. 93 (3), 228–238. 10.1124/mol.117.110841 29138268

[B49] Callahan B. J. McMurdie P. J. Rosen M. J. Han A. W. Johnson A. J. A. Holmes S. P. (2016). DADA2: High-Resolution Sample Inference From Illumina Amplicon Data. Nat. Methods 13 (7), 581–583. 10.1038/nmeth.3869 27214047 PMC4927377

[B9] Chen M. Hou P. Zhou M. Ren Q. Wang X. Huang L. (2020). Resveratrol Attenuates High-Fat Diet-Induced Non-alcoholic Steatohepatitis by Maintaining Gut Barrier Integrity and Inhibiting Gut Inflammation through Regulation of the Endocannabinoid System. Clin. Nutr. 39 (4), 1264–1275. 10.1016/j.clnu.2019.05.020 31189495

[B10] Contreras M. Ceric F. Torrealba F. (2007). Inactivation of the Interoceptive Insula Disrupts Drug Craving and Malaise Induced by Lithium. Science 318 (5850), 655–658. 10.1126/science.1145590 17962567

[B11] Di Marzo V. (2018). New Approaches and Challenges to Targeting the Endocannabinoid System. Nat. Rev. Drug Discov. 17 (9), 623–639. 10.1038/nrd.2018.115 30116049

[B12] Donvito G. Piscitelli F. Muldoon P. Jackson A. Vitale R. M. D'Aniello E. (2019). N-Oleoyl-glycine Reduces Nicotine Reward and Withdrawal in Mice. Neuropharmacology 148, 320–331. 10.1016/j.neuropharm.2018.03.020 29567093 PMC6408981

[B13] Fan Y. Ya-E Z. Ji-Dong W. Yu-Fan L. Ying Z. Ya-Lun S. (2018). Comparison of Microbial Diversity and Composition in Jejunum and colon of the Alcohol-dependent Rats. J. Microbiol. Biotechnol. 28 (11), 1883–1895. 10.4014/jmb.1806.06050 30270610

[B14] Gellert V. F. Holtzman S. G. (1978). Development and Maintenance of Morphine Tolerance and Dependence in the Rat by Scheduled Access to Morphine Drinking Solutions. J. Pharmacol. Exp. Ther. 205 (3), 536–546. 566320

[B15] Gicquelais R. E. Bohnert A. S. B. Thomas L. Foxman B. (2020). Opioid Agonist and Antagonist Use and the Gut Microbiota: Associations Among People in Addiction Treatment. Sci. Rep. 10 (1), 19471. 10.1038/s41598-020-76570-9 33173098 PMC7655955

[B16] Grazia Cascio M. Minassi A. Ligresti A. Appendino G. Burstein S. Di Marzo V. (2004). A Structure-Activity Relationship Study on N-Arachidonoyl-Amino Acids as Possible Endogenous Inhibitors of Fatty Acid Amide Hydrolase. Biochem. Biophys. Res. Commun. 314 (1), 192–196. 10.1016/j.bbrc.2003.12.075 14715265

[B17] Gruber A. J. McDonald R. J. (2012). Context, Emotion, and the Strategic Pursuit of Goals: Interactions Among Multiple Brain Systems Controlling Motivated Behavior. Front. Behav. Neurosci. 6, 50. 10.3389/fnbeh.2012.00050 22876225 PMC3411069

[B18] Guida F. Turco F. Iannotta M. De Gregorio D. Palumbo I. Sarnelli G. (2018). Antibiotic-induced Microbiota Perturbation Causes Gut Endocannabinoidome Changes, Hippocampal Neuroglial Reorganization and Depression in Mice. Brain Behav. Immun. 67, 230–245. 10.1016/j.bbi.2017.09.001 28890155

[B19] Huang S. M. Bisogno T. Petros T. J. Chang S. Y. Zavitsanos P. A. Zipkin R. E. (2001). Identification of a New Class of Molecules, the Arachidonyl Amino Acids, and Characterization of One Member that Inhibits Pain. J. Biol. Chem. 276 (46), 42639–42644. 10.1074/jbc.M107351200 11518719

[B20] Iannotti F. A. Di Marzo V. (2021). The Gut Microbiome, Endocannabinoids and Metabolic Disorders. J. Endocrinol. 248 (2), R83–R97. 10.1530/JOE-20-0444 33337346

[B21] Kang M. Mischel R. A. Bhave S. Komla E. Cho A. Huang C. (2017). The Effect of Gut Microbiome on Tolerance to Morphine Mediated Antinociception in Mice. Sci. Rep. 7, 42658. 10.1038/srep42658 28211545 PMC5314392

[B22] Katsidoni V. Kastellakis A. Panagis G. (2013). Biphasic Effects of Δ9-tetrahydrocannabinol on Brain Stimulation Reward and Motor Activity. Int. J. Neuropsychopharmacol. 16 (10), 2273–2284. 10.1017/S1461145713000709 23830148

[B23] Koob G. F. Maldonado R. Stinus L. (1992). Neural Substrates of Opiate Withdrawal. Trends Neurosci. 15 (5), 186–191. 10.1016/0166-2236(92)90171-4 1377426

[B24] Limebeer C. L. Rock E. M. Sharkey K. A. Parker L. A. (2018). Nausea-Induced 5-HT Release in the Interoceptive Insular Cortex and Regulation by Monoacylglycerol Lipase (MAGL) Inhibition and Cannabidiol. eNeuro 5 (4). 10.1523/ENEURO.0256-18.2018 PMC6071201 30073198

[B25] Llorente-Berzal A. Terzian A. L. di Marzo V. Micale V. Viveros M. P. Wotjak C. T. (2015). 2-AG Promotes the Expression of Conditioned Fear *via* Cannabinoid Receptor Type 1 on GABAergic Neurons. Psychopharmacology (Berl) 232 (15), 2811–2825. 10.1007/s00213-015-3917-y 25814137

[B26] Lyvers M. Yakimoff M. (2003). Neuropsychological Correlates of Opioid Dependence and Withdrawal. Addict. Behav. 28 (3), 605–611. 10.1016/S0306-4603(01)00253-2 12628632

[B27] Maione S. De Petrocellis L. de Novellis V. Moriello A. S. Palazzo E. (2007). Analgesic Actions of N-Arachidonoyl-Serotonin, a Fatty Acid Amide Hydrolase Inhibitor with Antagonistic Activity at Vanilloid TRPV1 Receptors. Br. J. Pharmacol. 150 (6), 766–781. 10.1038/sj.bjp.0707145 17279090 PMC2013858

[B28] Manca C. Boubertakh B. Leblanc N. Deschênes T. Lacroix S. Martin C. (2020a). Germ-free Mice Exhibit Profound Gut Microbiota-dependent Alterations of Intestinal Endocannabinoidome Signaling. J. Lipid Res. 61 (1), 70–85. 10.1194/jlr.RA119000424 31690638 PMC6939599

[B29] Manca C. Shen M. Boubertakh B. Martin C. Flamand N. Silvestri C. (2020b). Alterations of Brain Endocannabinoidome Signaling in Germ-free Mice. Biochim. Biophys. Acta Mol. Cel Biol Lipids 1865 (12), 158786. 10.1016/j.bbalip.2020.158786 32795503

[B30] Marsicano G. Wotjak C. T. Azad S. C. Bisogno T. Rammes G. Cascio M. G. (2002). The Endogenous Cannabinoid System Controls Extinction of Aversive Memories. Nature 418 (6897), 530–534. 10.1038/nature00839 12152079

[B31] Meckel K. R. Kiraly D. D. (2019). A Potential Role for the Gut Microbiome in Substance Use Disorders. Psychopharmacology (Berl) 236 (5), 1513–1530. 10.1007/s00213-019-05232-0 30982128 PMC6599482

[B32] Micale V. Cristino L. Tamburella A. Petrosino S. Leggio G. M. Drago F. (2009). Anxiolytic Effects in Mice of a Dual Blocker of Fatty Acid Amide Hydrolase and Transient Receptor Potential Vanilloid Type-1 Channels. Neuropsychopharmacology 34 (3), 593–606. 10.1038/npp.2008.98 18580871

[B33] Naqvi N. H. Bechara A. (2010). The Insula and Drug Addiction: An Interoceptive View of Pleasure, Urges, and Decision-Making. Brain Struct. Funct. 214 (5-6), 435–450. 10.1007/s00429-010-0268-7 20512364 PMC3698865

[B34] Paulson J. N. Pop M. Bravo H. C. (2018). MetagenomeSeq: Statistical Analysis for Sparse High-Throughput Sequencing. College Park, Maryland: Bioconductor. Available at: http://cbcb.umd.edu/software/metagenomeSeq .

[B35] Petrie G. N. Wills K. L. Piscitelli F. Smoum R. Limebeer C. L. Rock E. M. (2019). Oleoyl glycine: Interference with the Aversive Effects of Acute Naloxone-Precipitated MWD, but Not Morphine Reward, in Male Sprague-Dawley Rats. Psychopharmacology (Berl) 236 (9), 2623–2633. 10.1007/s00213-019-05237-9 30993360

[B36] Piscitelli F. Guida F. Luongo L. Iannotti F. A. Boccella S. Verde R. (2020). Protective Effects of N-Oleoylglycine in a Mouse Model of Mild Traumatic Brain Injury. ACS Chem. Neurosci. 11 (8), 1117–1128. 10.1021/acschemneuro.9b00633 32017529

[B37] Raboune S. Stuart J. M. Leishman E. Takacs S. M. Rhodes B. Basnet A. (2014). Novel Endogenous N-Acyl Amides Activate TRPV1-4 Receptors, BV-2 Microglia, and Are Regulated in Brain in an Acute Model of Inflammation. Front. Cell Neurosci. 8, 195. 10.3389/fncel.2014.00195 25136293 PMC4118021

[B38] Rubio M. Fernández-Ruiz J. de Miguel R. Maestro B. Michael Walker J. Ramos J. A. (2008). CB_1_ Receptor Blockade Reduces the Anxiogenic-like Response and Ameliorates the Neurochemical Imbalances Associated with Alcohol Withdrawal in Rats. Neuropharmacology 54 (6), 976–988. 10.1016/j.neuropharm.2008.02.005 18371990

[B50] Rock E. M. Ayoub S. M. Limebeer C. L. Gene A. Wills K. L. DeVuono M. V. (2020). Acute Naloxone-Precipitated Morphine Withdrawal Elicits Nausea-Like Somatic Behaviors in Rats in a Manner Suppressed by N-Oleoylglycine. Psychopharmacology 237 (2), 375–384. 10.1007/s00213-019-05373-2 31712968

[B39] Salavrakos M. Leclercq S. De Timary P. Dom G. (2021). Microbiome and Substances of Abuse. Prog. Neuropsychopharmacol. Biol. Psychiatry 105, 110113. 10.1016/j.pnpbp.2020.110113 32971216

[B40] Sharkey K. A. Cristino L. Oland L. D. Van Sickle M. D. Starowicz K. Pittman Q. J. (2007). Arvanil, Anandamide and N-Arachidonoyl-Dopamine (NADA) Inhibit Emesis through Cannabinoid CB1 and Vanilloid TRPV1 Receptors in the Ferret. Eur. J. Neurosci. 25 (9), 2773–2782. 10.1111/j.1460-9568.2007.05521.x 17459108

[B41] Sticht M. A. Limebeer C. L. Rafla B. R. Abdullah R. A. Poklis J. L. Ho W. (2016). Endocannabinoid Regulation of Nausea Is Mediated by 2-arachidonoylglycerol (2-AG) in the Rat Visceral Insular Cortex. Neuropharmacology 102, 92–102. 10.1016/j.neuropharm.2015.10.039 26541329 PMC4698202

[B42] Sticht M. A. Limebeer C. L. Rafla B. R. Parker L. A. (2015). Intra-visceral Insular Cortex 2-arachidonoylglycerol, but Not N-Arachidonoylethanolamide, Suppresses Acute Nausea-Induced Conditioned Gaping in Rats. Neuroscience 286, 338–344. 10.1016/j.neuroscience.2014.11.058 25499318

[B43] Sulcova E. Mechoulam R. Fride E. (1998). Biphasic Effects of Anandamide. Pharmacol. Biochem. Behav. 59 (2), 347–352. 10.1016/s0091-3057(97)00422-x 9476980

[B44] Thomaz A. C. Iyer V. Woodward T. J. Hohmann A. G. (2021). Fecal Microbiota Transplantation and Antibiotic Treatment Attenuate Naloxone-Precipitated Opioid Withdrawal in Morphine-dependent Mice. Exp. Neurol. 343, 113787. 10.1016/j.expneurol.2021.113787 34153321 PMC8477666

[B45] Wang F. Meng J. Zhang L. Johnson T. Chen C. Roy S. (2018). Morphine Induces Changes in the Gut Microbiome and Metabolome in a Morphine Dependence Model. Sci. Rep. 8, 3596. 10.1038/s41598-018-21915-8 29483538 PMC5827657

[B46] Wills K. L. Parker L. A. (2016). Effect of Pharmacological Modulation of the Endocannabinoid System on Opiate Withdrawal: A Review of the Preclinical Animal Literature. Front. Pharmacol. 7, 187. 10.3389/fphar.2016.00187 27445822 PMC4923145

[B47] Wills K. L. Petrie G. N. Millett G. Limebeer C. L. Rock E. M. Niphakis M. J. (2016). Double Dissociation of Monoacylglycerol Lipase Inhibition and CB1 Antagonism in the Central Amygdala, Basolateral Amygdala, and the Interoceptive Insular Cortex on the Affective Properties of Acute Naloxone-Precipitated Morphine Withdrawal in Rats. Neuropsychopharmacology 41 (7), 1865–1873. 10.1038/npp.2015.356 26647976 PMC4869055

[B48] Zhang L. Meng J. Ban Y. Jalodia R. Chupikova I. Fernandez I. (2019). Morphine Tolerance Is Attenuated in Germfree Mice and Reversed by Probiotics, Implicating the Role of Gut Microbiome. Proc. Natl. Acad. Sci. U S A. 116 (27), 13523–13532. 10.1073/pnas.1901182116 31209039 PMC6613141

