# Supplementary figures and images for: Spontaneous and Naloxone-Precipitated Withdrawal Behaviors From Chronic Opiates are Accompanied by Changes in N-Oleoylglycine and N-Oleoylalanine Levels in the Brain and Ameliorated by Treatment With These Mediators
Source: Front Pharmacol. 2021 Sep 15;12:706703. doi: 10.3389/fphar.2021.706703 (PMC8479102; doi:10.3389/fphar.2021.706703)

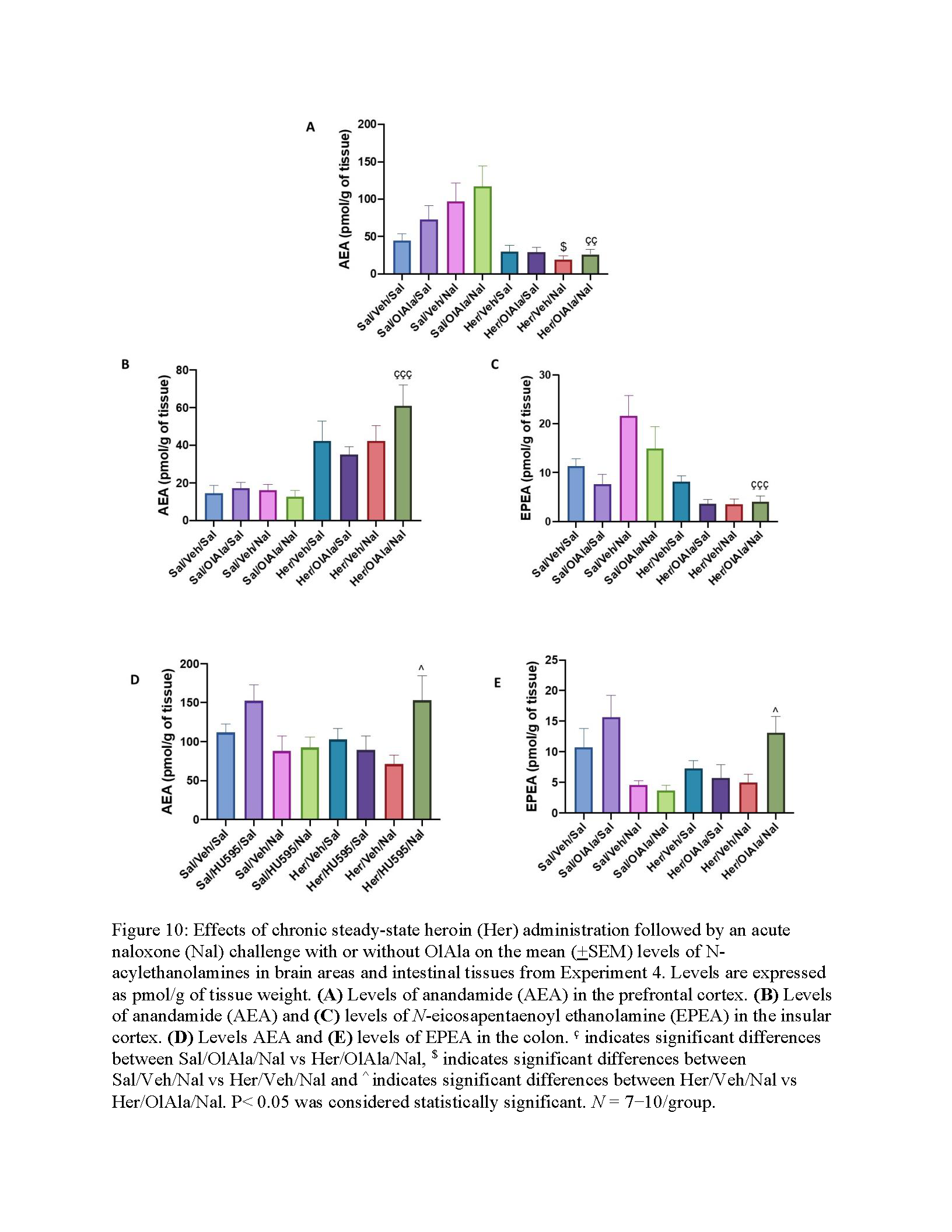

Supplement: Supplementary file 1 [file Image1.tiff]
